# Advancements in Technologies Targeting Horizontal Gene Transfer—Routes to Control Drug Resistance Evolution

**DOI:** 10.1021/acsbiomedchemau.5c00273

**Published:** 2026-04-09

**Authors:** Samuel Chetachukwu Adegoke, Md Adnan Karim, Maurelio Cabo Jr, Ignatius Senyo Yao Yawlui, Dennis LaJeunesse

**Affiliations:** Department of Nanoscience, Joint School of Nanoscience and Nanoengineering, E Gate City Blvd, Greensboro, North Carolina 27401, United States

**Keywords:** multidrug resistance, natural transformation, resistance evolution, horizontal gene transfer, extended-spectrum β-lactamases (ESBLs), advance oxidation process (AOP), competence blockers, gene drives, antiplasmids systems

## Abstract

The global rise of multidrug-resistant (MDR) bacteria poses a major public health crisis, threatening the effectiveness of modern medicine. Traditional antibiotic development struggles to keep pace with bacterial evolution, largely due to the rapid dissemination of antibiotic resistance genes via horizontal gene transfer (HGT). HGT mechanisms both canonical and noncanonical enable bacteria to acquire resistance traits defining species and even special challenges. In this review, we cover the current understanding of HGT in spreading antibiotic resistance and explore possible strategies to control HGT and slow the spread of antimicrobial resistance. Recent advances highlight the potential of synthetic competence inhibitors, advanced oxidation processes (AOPs), CRISPR-Cas technologies, gene drives, and antiplasmids to disrupt horizontal gene flow and mitigate resistance evolution. Despite promising laboratory results, challenges remain in translating these approaches into clinical and environmental applications. Blocking HGT could complement antimicrobial stewardship programs and traditional antibiotic therapies by curbing the emergence of new resistant strains at their genetic roots. By targeting the foundational mechanisms of resistance acquisition, these strategies offer a proactive pathway to extend the efficacy of existing antibiotics and prevent a “postantibiotic” era. Ongoing research into bacterial pathogenesis, genome defense systems, and innovative gene-editing technologies will be critical to developing effective, scalable solutions for managing MDR infections worldwide.

## Graphical abstract

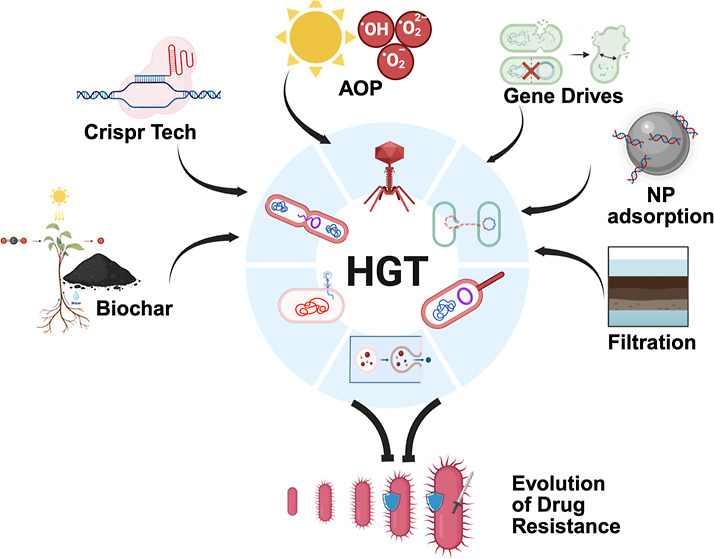

## 1 Introduction

Bacterial multidrug resistance (MDR) is an urgent global public health crisis, accounting for over 700,000 deaths per year and potentially reaching 10 million by 2050.^1–3^ While antibiotics play a vital role in disease prevention and management, their effectiveness is imperiled by the emergence of resistant strains.^1^ The evolution of resistance and virulent strains of pathogenic bacteria have culminated in a huge setback for the development of new classes of antibiotics. Despite advancements in antibiotic discovery, identifying new antibiotics against established or newly identified essential bacterial targets remains difficult.^4^ Molecular evolution, which involves DNA sequence alterations, including mutations and other changes, can impact gene products or expression, potentially resulting in functional loss, enhancement, or no significant effect.

We are entering a “postantibiotic era”, where formerly manageable infections once again transform into life-threatening conditions due to ineffective drugs.^5^ Due to the ongoing evolution of multidrug resistance, many first-line antibiotics have become ineffective. While the introduction of new antibiotic drugs initially slowed rising resistance levels, decades of research indicate that merely restricting antibiotic usage cannot reliably reverse the evolution of resistance. In response to the evolution of resistance, newer approaches that combined antibiotics with specific resistance-inhibiting compounds have successfully improved and extended the utility of certain antibacterial agents.^6^ However, these newer protocols still fail to prevent selective pressures that lead to the evolution of resistance over longer time scales.^1^ The problem resides in the fact that bacteria will evolve resistance to any drug and that once resistance has evolved it will be shared with the entire microbiome community. Prokaryotic horizontal gene transfer (pHGT) drives the dissemination of antibiotic resistance and the genesis of new pathogenic bacterial strains. Selective pressures such as the indiscriminate use of antibiotics create the environment necessary for the evolution of drug resistance. Once these advantageous genetic elements evolve in one species, natural transformation ensures that they will be shared with the entire microbiome community. While horizontal gene transfer (HGT) is recognized as a key evolutionary mechanism in acquired resistance, recent evidence indicates antibiotic use dramatically accelerates its rate, potentially precipitating clinical failure over the course of a single infection.^4^ This review examines horizontal gene transfer and the prospect of slowing the spread of bacterial multidrug resistance by blocking horizontal gene transfer. Specifically, we explore the possibilities of blocking horizontal DNA exchange as a strategy to avert the emergence of new resistant strains. Although intriguing scientifically, these approaches for combatting the spread of resistance evolution are relatively nascent and remain novel regarding real-world applications. However, we anticipate progress in molecular biology and understanding in bacterial pathogenesis will lead to future opportunities for such strategies, particularly as traditional antibiotics grow increasingly obsolete in the face of mounting resistance.

### 1.1 Genetic Elements Associated with Bacterial HGT

HGT serves as the principal route of evolution and represents a critical challenge for healthcare globally.^7^ Bacterial survival and adaptation are intrinsically linked to the genetic materials that they acquire through both canonical and noncanonical HGT pathways. Central to the mechanisms of HGT are the genetic elements that carry new information within emerging populations of bacteria. There are two classes of genetic elements, mobile genetic elements (MGEs) and MGE-independent elements (MGEIs) (Table 1). MGEs encode and/or carry the machinery required to replicate and mobilize DNA across cells and include plasmids, integrative and conjugative elements (ICEs), bacteriophages, and transposons and integrons that hitchhike on plasmids/ICE.^8–10^ MGEs are essential components of canonical HGT processes like conjugation and transduction and have played essential roles in the development of molecular biology and biotechnology as well.^9^ Alternatively, MGEIs are genetic elements that do not carry genes required for self-replication and/or self-mobilization. The MGEIs material includes the free environmental DNA that is either secreted from livings cells in processes like biofilm formation or DNA released from cells that have ruptured.^11^ MGE-independent genetic materials are often collected from the environment by the canonical HGT process known as natural transformation. Furthermore, some mechanisms like nanotube-mediated DNA exchange and bacterial extracellular vesicles (OMVs, MVs, and O-IMVs) involve both MGE-dependent and MGE-independent elements.^12,13^

**Table 1:** MGE versus MGE-Independent Pathways for HGT

pathway type	mechanisms	examples	key features
MGE-dependent	plasmids (conjugation), ICEs, transposons, bacteriophages (transduction), GTAs	*E. coli* (plasmid transfer), Vibrio cholerae (ICE), Salmonella (phage-mediated)	requires mobile genetic elements; often encodes transfer machinery; efficient and directional
MGE-independent	natural transformation (uptake of free DNA), nanotube-mediated exchange, extracellular vesicles (OMVs, MVs, and O-IMVs)	Acinetobacter baylyi (transformation), *Bacillus subtilis* (nanotubes), Pseudomonas aeruginosa (OMVs)	does not rely on self-mobilizing elements; opportunistic; influenced by environmental conditions

### 1.2 Classes of Genetic Elements in HGT

There are several classes of genetic elements that are involved in HGT (Table 2). Plasmids are extrachromosomal DNA molecules that replicate independently in the bacteria and often carry genes for antibiotic resistance or virulence.^14^ Structurally, most plasmids are circular, but some like the 143kb pELF1 in *Enterococcus faecium*, which carries a vancomycin resistance gene, is linear.^15,16^ In HGT, plasmids are primarily transferred via conjugation but have also been demonstrated also to be transferred between cells via nanotubes or bacterial extracellular vesicles (bEVs).^17^ Plasmids range in size from 10kb up to 100kb with some larger (>100kb) megaplasmids in some pathogenic bacteria.^18,19^ All plasmids carry an origin of replication (ori), which is essential for their autonomous replication within a host cell.^18^ Examples of plasmids that have been identified as carriers of antibiotic resistance genes include Inc.F plasmids which are common in *Enterobacteriaceae* and carry ESBL genes like bla_CTX‑M_, bla_TEM_, and bla_SHV_; ncI plasmids which are frequently associated with bla_CTX‑M_ and other β-lactamase genes; ncP plasmids including RP4, which are broad-host-range plasmids that carry multiple resistance genes, including tet, sul, and bla; Inc.A/C plasmids, which are found in multidrug-resistant *Salmonella* spp. and *Escherichia coli* (*E. coli*) and carry genes for β-lactams, aminoglycosides, and sulfonamides; Inc.X plasmids which are associated with bla_KPC_ and mcr-1 in colistin resistance; and pNDM plasmids which carry bla_NDM_ (New Delhi metallo-β-lactamase and conferring carbapenem resistance).^16,20–24^

**Table 2:** Genetic Elements Involved in Horizontal Gene Transfer in Bacteria

Genetic Element	Mobile genetic element (Y/N)	Associated HGT Mechanism	Examples
plasmids	yes	conjugation, nanotube-mediated transfer	pNDM, Inc.F, Inc.A/C, Inc. X, Inc.P^16,20–22,60^
bacteriophages	yes	transduction (generalized, specialized, lateral)	ΦCTX, Staphylococcus aureus phages, Enterococcus faecalis phages, Salmonella spp. and E. coli phages, environmental phages^28–30,61^
integrative and conjugative elements (ICEs)	yes	conjugation	Tn916 family ICE elements, ICESt1, ICESt, ICEBs1, SXT/R391 family ICEs, ICEclc^33–35,51^
transposons	yes	mobilization via plasmids or ICEs	Tn2, Tn3, Tn21, Tn7, Tn1546, Tn125,^39,45,47–49^
free environmental DNA	no	natural transformation	*Streptococcus pneumoniae*, *Neisseria gonorrheae*

Bacteriophages are another MRE genetic element that mediates the delivery of new genetic information to a recipient cell. Bacteriophages are bacterial viruses with genomes that range in size from 3 kb to >500 kb.^25^ The genetic material of a bacteriophage can be either DNA or RNA and encodes structural genes, i.e., capsid proteins and tail fibers, genes required for replication, e.g., DNA polymerase, regulatory genes that control lytic vs lysogenic cycles, and accessory genes. Bacteriophages can also transfer chromosomal fragments or resistance genes between bacteria.^26^ Examples of bacteriophages that transfer ARGs include ΦCTX which carries ctxAB genes in *Vibrio cholerae* (*V. cholerae)*, the cholera toxin genes, but not antibiotic resistance but demonstrates phage-mediated virulence gene transfer; *Staphylococcus aureus* (*S. aureus)* phage, which carry mecA and fusB genes, contributing to methicillin and fusidic acid resistance; *Enterococcus faecalis* phages which carry tet­(M) and erm­(B) genes, conferring tetracycline and macrolide resistance; *Salmonella* and *E. coli* phage, which have been implicated in carrying bla_CTX‑M_ and qnr genes, aiding β-lactam and fluoroquinolone resistance; and various undescribed environmental phage which in metagenomic studies show ARGs like sul, tet, and bla in phage DNA from wastewater and soil.^27–30^

Integrative and conjugative elements (ICEs) are a large family of mobile genetic elements with two defining features: they are integrated into a host genome, and they encode a functional conjugation system, a type IV secretion system.^31^ These two qualities enable ICEs to be transferred to other cells and spreading adaptive traits like drug resistance or enhanced virulence. Examples of ICEs that transfer ARGs include Tn916 Family that carry tet (M) resistance in *Enterococcus*, *Streptococcus*, and *Clostridium* spp; ICESt1 and ICESt that carry erm­(B) encoding macrolide resistance in *Streptococcus thermophilus*; ICEBs1, which carry tetracycline and macrolide resistance genes when mobilized in *Bacillus subtilis*; SXT/R391 Family ICEs carrying sul, dfrA, and str (aka, sulfonamide, trimethoprim, and streptomycin resistance) in *V. cholerae*, *Proteus* ssp, *Providencia* ssp.; and ICEclc which has been linked to multidrug resistance gene in *Pseudomonas knackmussii*.^32–36^

Transposons are mobile genetic elements that can move from one DNA location to another through cut-and-paste or copy-and-paste transposition mechanisms driven by a transposase (tnp) enzyme.^37–39^ Many transposons carry an additional genetic element including integrons, antibiotic resistance genes, or other cargo as they move, allowing them to disseminate resistance traits across genomes and between bacterial species.^38^ Integrons are genetic platforms that capture, integrate, and express gene cassettes often including antibiotic resistance genes through a site-specific recombination system mediated by an integrase gene^40–43^ (Figure 4). While integrons are not mobile, they are frequently embedded within mobile elements such as transposons or plasmids, which enable their horizontal transfer between bacteria.^44,45^ Transposons often hitchhike on plasmids or ICEs. Several classes of transposons have been observed to carry ARGs. The Tn3 transposon has been shown to be a common carrier in *E. coli* and many *Enterobacteriaceae* spp. of blaTEM-1 (β-lactamase) resistance genes via conjugation plasmids.^39,46^ Tn21, a Class 1 integron-associated transposon, has a broad range of activity in Gram-negative bacteria and is one of the most globally distributed multidrug resistance transposon due to its integron.^45^ Tn7 transposons that carry the trimethoprim resistance dfrA1 gene in *E. coli*, *Salmonella* ssp., and *Pseudomonas* ssp. integrate at a specific chromosomal site (attTn7) and often carry integron structures.^47,48^ The opportunistic pathogens *E. faecium* and *E. faecalis* which are responsible for many serious hospital-acquired infections including urinary tract infections, bacteremia (bloodstream infections), and infective endocarditis often carry the vanA vancomycin resistance gene on a Tn1546 transposon which can be found on conjugative plasmids.^49^ The Tn4401 and Tn125 transposon is the primary driver of carbapenems worldwide resistance in *Klebsiella pneumoniae*, *Acinetobacter baumannii*, and other *Enterobacteriaceae* bacteria.^39,50,51^

**Figure 1:**
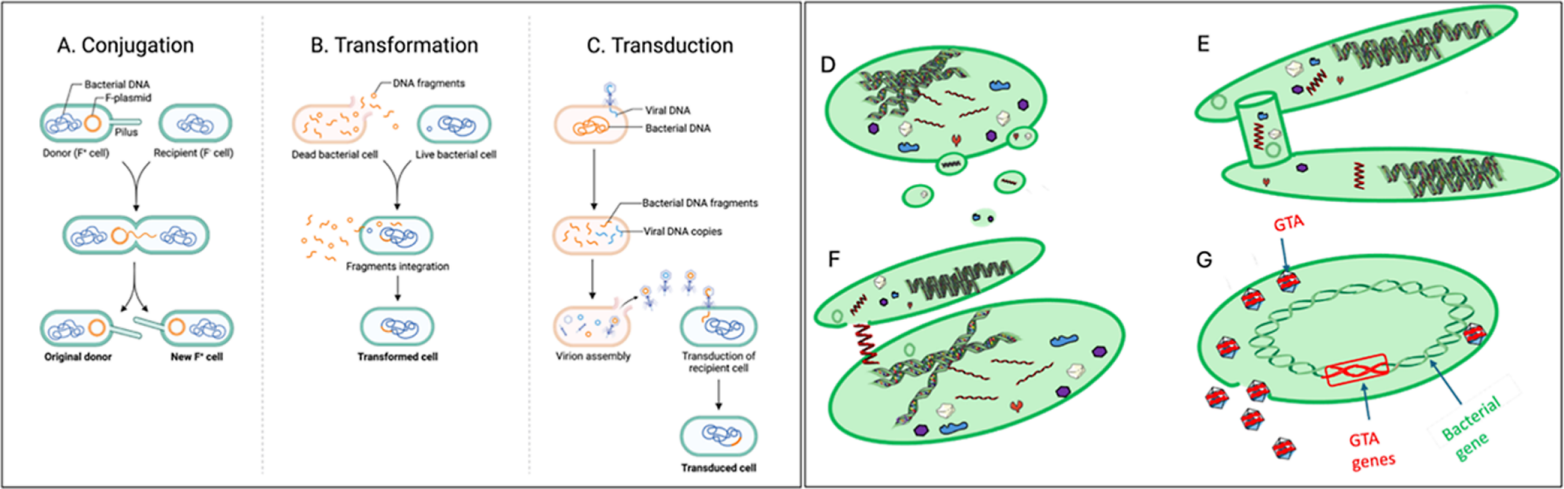
Canonical and noncanonical routes of HGT. (A) Conjugation involves cell-to-cell contact. (B) Transformation involves the uptake of extracellular DNA from the environment. (C) Transduction involves attachment of phage to bacteria followed by subsequent injection of its DNA into the host. (D) HGT through membrane vesicles, where bacteria release vesicles containing cellular materials that are taken up by recipient cells. (E) HGT through nanotubes, which are membrane extensions forming direct bridges between neighboring bacterial cells to enable exchange of cytoplasmic materials. (F) HGT through autolysis. Autolysis involves the self-lysis of a subpopulation of bacterial cells, releasing extracellular DNA that becomes available for uptake by naturally competent bacterial cells. (G) HGT via gene transfer agents (GTAs), which package host DNA fragments and release them upon lysis for uptake by recipient cells.

**Figure 2:**
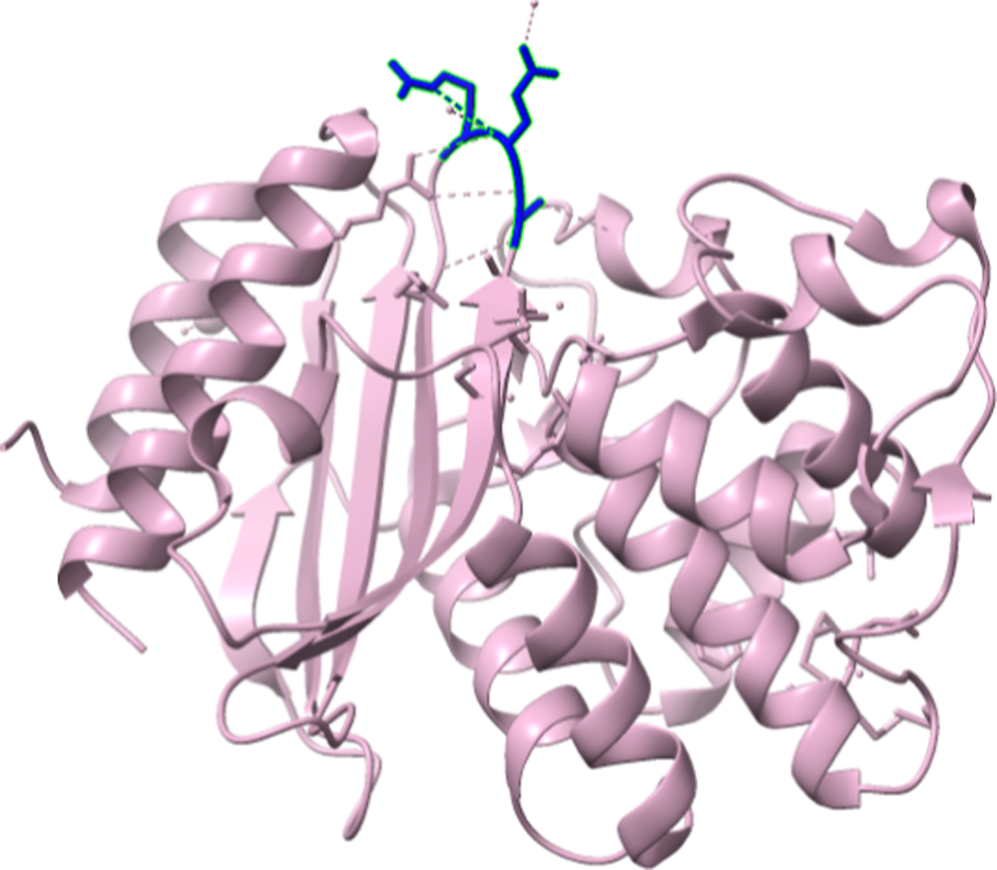
Crystal structure of stabilized TEM-1 beta-lactamase variant v.13 carrying G238S mutation.^129^ The blue colored region with hydrogen bonding is the mutation region.

**Figure 3:**
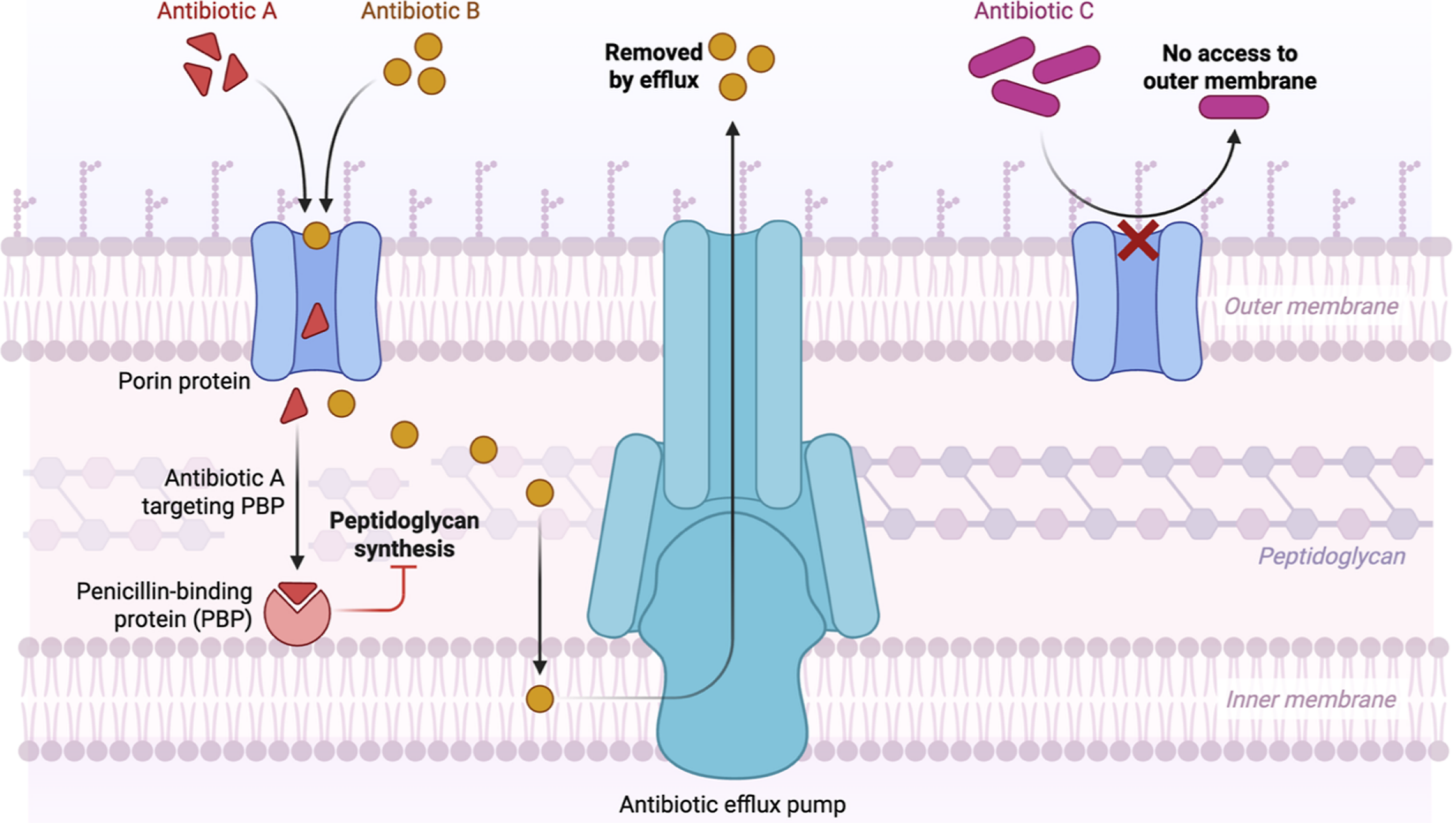
Schematic representation of the efflux pump system in the Gram-negative bacterial cell membrane. The outer membrane (OM) at the top, the peptidoglycan layer (PGL) in the middle, and the inner membrane (IM) at the bottom, with the cytoplasm beneath. Porins are water-filled channels embedded in the outer membrane that allow hydrophilic molecules and small metabolites to pass through passively. They are essential for nutrient uptake and waste expulsion, functioning as molecular sieves that exclude larger or hydrophobic molecules.

**Figure 4:**
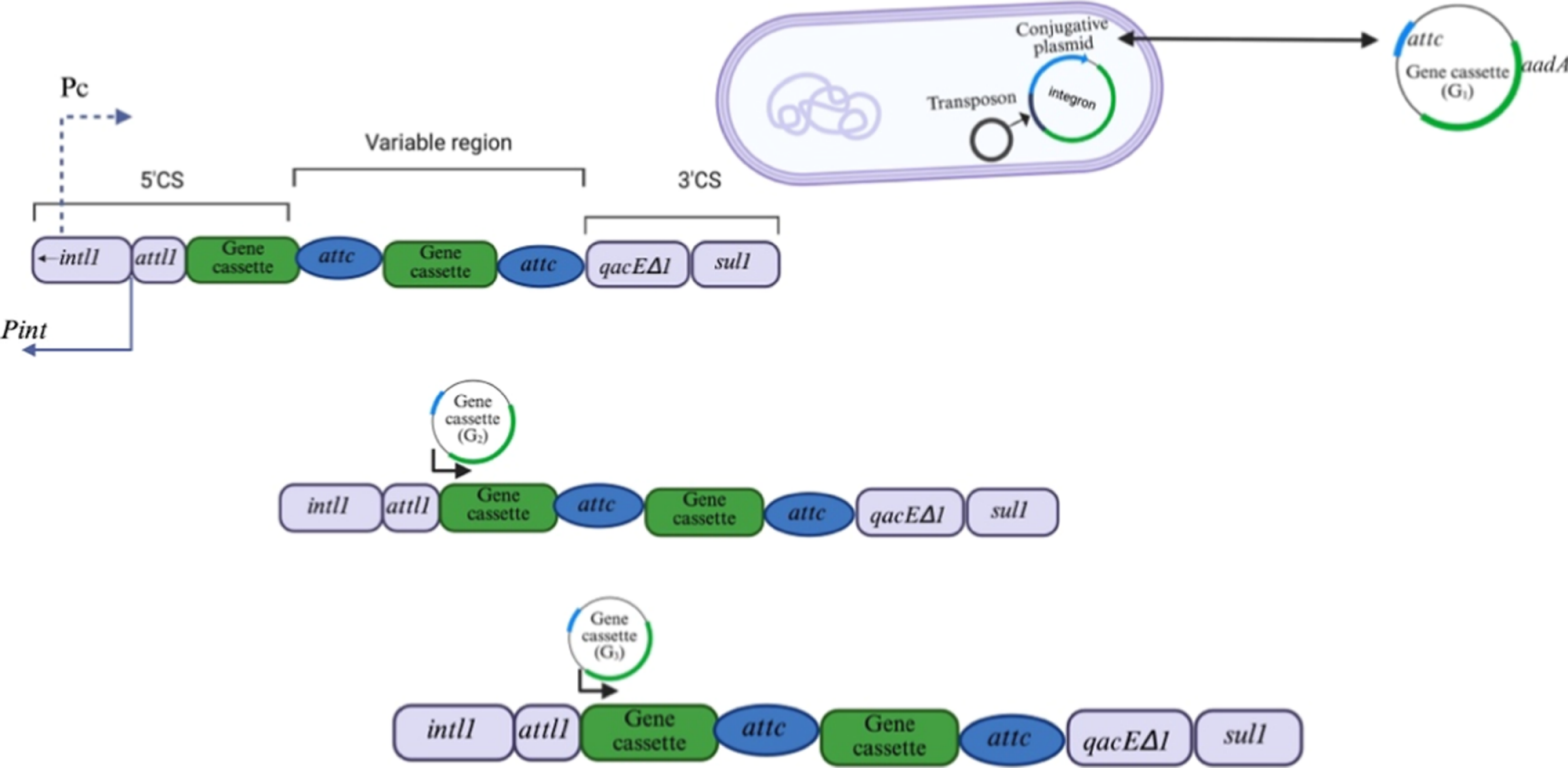
Structure of class 1 integrons. Integrons function as modular genetic elements that facilitate the acquisition, chromosomal insertion, and expression of gene cassettes, frequently encoding antibiotic resistance determinants, via a site-specific recombination mechanism catalyzed by an integrase enzyme.

Environmental DNA can be taken up by bacteria during natural transformation. These events often allow *Streptococcus pneumoniae* and *Neisseria gonorrheae* acquire free DNA fragments containing altered resistance to several classes of antibiotics.^52–57^ In many cases, the generation of mosaic genes such as penicillin-binding protein (PBP) genes is a result of recombination events conferring high-level penicillin resistance.^58,59^

### 1.3 Canonical Horizontal Gene Transfer (HGT): The Big Three

There are several mechanisms by which horizontal gene transfer is perpetuated in bacterial ecosystems. The three canonical forms of HGT are transformation, transduction, and conjugation which have been extensively studied (Table 3).

**Table 3:** Three Types of Bacterial Transduction

Type of Transduction	Mechanism	Key Features	Example
generalized	random bacterial DNA fragments are accidentally packaged into phage capsids during the lytic cycle.	transfers any gene; occurs at low frequency.	Salmonella ssp., E. coli
specialized	prophage excises incorrectly from host chromosome, taking adjacent bacterial genes with it.	transfers only genes near integration site.	λ phage in E. coli (gal, bio)
lateral/progressive	phage replicates while integrated, packaging large contiguous regions of bacterial DNA.	transfers large chromosomal segments; newly discovered.	Salmonella, Staphylococcus aureus

#### 1.3.1 Transformation

There are two types of transformation: engineered/artificial transformation, which is a laboratory technique used to introduce foreign DNA into bacterial cells by creating temporary permeability in the cell membrane and natural transformation which is the focus of this section. Natural transformation is the ability of a bacterium to take up exogenous/extracellular DNA.^62^ Transformation refers to the process by which bacteria take up and acquire exogenous DNA from their surrounding environment^7,63,64^ (Figure 1B). In 1928, the finding that bacteria undergo natural transformation set the stage for the landmark realization 16 years later that DNA acts as the genetic material encoding hereditary information.^7^ Transformation involves a translocation mechanism that is responsible for moving environmental/extracellular DNA across membranes. Of the four translocation systems found in Gram-negative bacteria, only the Class II type 4 pilus assembly systems (T4P) are essential for bacterial transformation.^65,66^ T4P systems share structural similarities with other Class 2 pili-based translocation systems such as the type 2 secretion system (T2SS) but differ by producing a pilus that is capable of dynamic extension and retraction; furthermore, T4P systems also play roles in motility.^67^ The T4P assembly system in Gram-negative bacteria is a highly dynamic system that is regulated by dedicated ATPases. Pilins initially reside in the membrane, with assembly driven by ATPase-mediated extraction of transmembrane helices, which then pack into filaments. While DNA uptake complexes are well characterized, the environmental conditions that promote pili-DNA binding remain unclear, particularly in Gram-negative species where transforming DNA must traverse two membranes and the periplasm. Natural transformation is influenced by intrinsic and extrinsic factors, including genotoxic stress, nutrient availability, DNA length, salt concentration, biofilm architecture, temperature, and starvation.^68^ Nutritional status also is a key driver of transformation.^68^ For instance, in *V. cholerae*, the causative agent of cholera, the polysaccharide chitin triggers the uptake of extracellular DNA.^68^ Moreover, other triggers have been observed including artificial sweeteners in the Gram-negative bacterium *Acinetobacter baylyi* ADP1.^69^ Despite natural transformation being an inherent capability of many bacteria, the number of bacterial species that demonstrate natural competence is limited at around 80 known species.^70,71^

#### 1.3.2 Transduction

Transduction involves the sharing of the genetic material mediated by bacterial viruses/bacteriophages.^72^ There are two main types of transduction mechanisms: generalized transduction, which occurs during the phage lytic cycle when nonphage genome/host DNA is mistakenly packaged in a new phage particle (Figure 1C). The resulting transducing phage released upon lysis can then transfer this new genetic material into another bacterium. The second type of transduction is called specialized transduction, which during the lysogenic phase, i.e., the integration of phage DNA into the host genome in dormancy only to excise adjacent sequences during the excision of the prophage whereupon new host sequences are incorporated into the phage particles and subsequently transferred to another host cell.^73–75^ Both forms of transduction have been implicated in the spread of drug resistance genes. Until recently, generalized transduction has been dismissed as too rare to be significant, but its role in the spread of drug resistance genes has been demonstrated by an elegant experiment in which two strains of methicillin-resistant *S. aureus* containing two distinct antibiotic resistance genes were cocultures and then infected with a generalized transducing phage resulting in the generation of double resistance after a short period of incubation^63,76,77^ (Figure 1C). Recently, a third type of transduction, called lateral transduction, has been identified in the temperate phages of *S. aureus*.^78^ Lateral transduction is a type of genetic transduction where large spans of DNA are transferred from one bacterium to another. This process is initiated by the packaging of DNA into transducing particles, which is facilitated by phage-mediated DNA transfer.^72^ In terms of frequency, lateral transduction is highly efficient. It has been observed that the regions of DNA transferred through lateral transduction are significantly larger than those transferred by classical mobile genetic elements.^79^ Lateral/progressive transduction is inherent to the life cycle of certain phages, such as the archetypical *Salmonella* phage P22. It allows these phages to balance propagation with lateral transduction, thereby enhancing their overall fitness.^80^ Unlike earlier occurrences, lateral transduction does not seem to stem from an erroneous process within the phage.^77^

#### 1.3.3 Conjugation

Conjugation involves the unidirectional transfer of DNA from a donor cell to a recipient cell through direct cell-to-cell contact.^78,81^ This HGT system involves a specific MRE, a conjugative plasmid or integrative conjugative element (ICEs) that encodes the machinery for pilus formation and DNA transfer.^63,78^ Furthermore, the shared DNA is actively processed and transferred through a type IV secretion systems.^63,78^ The various protein complexes that facilitate DNA transfer during bacterial conjugation are commonly encoded by conjugative plasmids or other mobile genetic elements such as ICEs (Figure 1A). Unlike other canonical HGT, conjugation is often species integrated by conjugative elements; however, conjugation can also impact a diverse population of bacteria too. In one study, using a broad-host-range RP4 conjugative plasmid carrying resistance to ampicillin, tetracycline, and kanamycin was shown to transfer from a *E. coli* donor to multiple recipient species in vitro.^82^ Conjugation is mediated by the mating pair formation (Mpf) system, which consists of ten conserved protein regions, that encode components of a DNA translocation channel including the extracellular conjugative pilus and a membrane-spanning protein complex.^83^ Together, these structures facilitate close cell-to-cell contact between the donor and recipient bacteria during conjugation. The Mpf complex also requires additional coupling protein (CP) that binds with the plasmid DNA substrate, enabling DNA transfer.^83^

Recipients included pathogenic and opportunistic bacteria such as *V. cholerae*, *Salmonella typhimurium*, *K. pneumoniae*, *Pseudomonas putida*, *Helicobacter pylori,* and *Agrobacterium tumefaciens* with transfer frequencies ranging from 10^–2^ to 10^–8^ depending on the donor–recipient combination (^43^). This study highlighted how conjugative plasmids like RP4 mediate horizontal gene transfer of multidrug resistance genes across diverse bacterial genera—including human pathogens—highlighting their significant role in the dissemination of antibiotic resistance. Within the donor bacterium, conjugation is a complex that requires multiple internal steps in the donor bacterium to prepare and execute plasmid transfer during conjugation. In Gram-negative bacteria, the genes encoded on the plasmids perform conjugative transfers including the transfer genes (tra) which encode components of the pili genes and other relevant elements such as the origin of transfer.^84^ Conjugation is tightly regulated by a myriad of factors including quorum sensing (QS), which is the cell-to-cell communication system used by bacteria to coordinate gene expression and behavior based on population density.^84–86^ A good example of inhibition of conjugation via QS is observed between the donor *E. coli* strain SM10λπ strain and the receiving bacterium *Pseudomonas aeruginosa*. In this example, activation of the SdiA gene which encodes a LuxR-type transcriptional regulator that plays a role in quorum sensing of *E. coli* binds the QS molecule N-Acyl-Homoserine Lactone produced by *P. aeruginosa,* thereby inhibiting the conjugation reaction between *E. coli* SM10λπ and the receiving *P. aeruginosa* cell by inhibiting the expression of tra genes.^64,78,87^

### 1.4 Noncanonical Horizontal Gene Transfer Agents—The New Kids on the Block

The canonical genetic transfer mechanisms—transformation, transduction, and conjugation—are well documented. However, emerging research identified new noncanonical gene transfer routes including Gene transfer agents (GTAs), bacterial extracellular vesicles (bEV), Nanotube-Mediated DNA exchange, and cell-to-cell natural transformation-mediated plasmid transfer (CTCNT-P)^13,88,89^ (Figure 1D–G).

#### 1.4.1 Gene Transfer Agents (GTAs)

GTAs are elements thought to be closely related to phages and have been observed in prokaryotes and archaea^90^ (Figure 1G). GTA production and release mechanism are still poorly characterized. These diminutive virus-like structures use a distinctive HGT pathway that bridges bacteriophage transduction and natural transformation. Although GTAs are common in the class alpha-proteobacteria from the order *Rhodobacterales*, in other bacteria, GTAs often carry MGE to the disseminate traits associated with virulence and antibiotic resistance.^88,90–92^ These noncanonical genetic elements are classified based on several factors including the ability of the MGE to transfer only its cargo genes or transfer its cargo and part of the hosts’ chromosomal DNA, which mirrors the behavior of bacteriophage transduction.^88^ While GTAs share similarity with generalized transduction, they differ in several ways. GTA-mediated transduction operates with higher efficiency compared to generalized transduction. Transducing phages operate primarily for self-propagation, while GTAs display no preferences for disseminating their own genes and are wholly dependent on the host for survival. The total lack of specificity in DNA packaging renders GTAs particularly compelling and raises significant questions about their influence on HGT, bacterial evolution, and the selective pressures maintaining their existence.^93^

Within a microbial population, the expression of GTAs is limited to only a portion of bacterial cells and is regulated by various factors including growth stage, phosphate concentration, quorum sensing regulators such as gafA, pleiotropic protein regulators such as CtrA, and cell cycle control proteins including CckA, DivL, and ChpT.^88,93^ However, the exact mechanisms that regulate the expression of GTAs remain unresolved. Recently, several genes required for GTA expression have been identified in two bacterial species, gafA in *Rhodobacter capsulatus*, and GafY and GafZ in *Caulobacter crescentus*; however, the products of these gene have no clear biochemical function making the mechanism still unclear.^93,94^

#### 1.4.2 Bacterial Extracellular Vesicles

Another noncanonical pathway for HGT is bacterial extracellular vesicles (bEVs) that have been identified in Gram-negative bacteria^12^ (Figure 1D). There are three classes of bEVs that are important for HGT: outer membrane vesicles (OMVs), membrane vesicles (MVs), and outer-inner membrane vesicles (O-IMVs)^12,95–98^ (Table 4). OMVs and MVs range in size from 20 to 250 nm, while O-IMVs are larger 100–450 nm and often contain cytoplasm and larger portion of chromosome,^99^ structurally OMV and MVs are single-membrane-layered structures, while O-IMVs are double bilayer structures being derived from inner and outer membranes.^99^ While the precise molecular mechanisms governing bEV biogenesis are currently being elucidated, recent work has shown that a combination of genetic determinants and perturbations in the membrane–peptidoglycan interface via cellular stress, peptidoglycan fragment accumulation, and/or periplasmic misfolded protein aggregation induces MVs biogenesis.^12,88,100^ Outer membrane vesicle (OMVs) are secreted by Gram-negative bacterial cells and participate in diverse biological functions including virulence factor delivery, metabolite secretion, bacteriophage infection resistance, intercellular communication, and host immune system modulation, in addition to their role in horizontal gene transfer.^12,101^ OMVs have been shown to deliver a variety of different biomolecule cargos including virulence genes, adhesion proteins, toxins, antibiotic resistance genes, immune modulating factors, and other nonspecific genetic materials.^12,102,103^ The last type of bEVs are O-IMVs which are also generated by Gram-negative bacteria and are larger than either MVs or OMVs due to their being derived from both inner and outer membranes.

**Table 4:** Bacterial Extracellular Vesicle (bEV) Horizontal Gene Transfer Pathways

Type	Size	Structure	Origin	Cargo	Role in HGT
outer membrane vesicles (OMVs)	∼20–250 nm	single lipid bilayer	Gram-negative outer membrane	DNA, RNA, proteins, toxins	deliver genetic material directly to recipient cells
membrane vesicles (MVs)	∼20–400 nm	single lipid bilayer	Gram-positive cytoplasmic membrane	chromosomal fragments, plasmids	facilitate gene transfer between Gram-positive bacteria
outer-inner membrane vesicles (O-IMVs)	∼100–450 nm (often larger than OMVs because they include cytoplasmic content)	double-layered structures	both inner and outer membranes of Gram-negative bacteria	cytoplasmic content including DNA	enable transfer of larger genetic elements and cytoplasmic molecules

#### 1.4.3 Nanotube-Mediated DNA Exchange

Nanotube-mediated DNA exchange is similar to conjugation as it also involves direct cell-to-cell contact.^104–106^ However, unlike conjugation which uses a proteinaceous tube, nanotube-mediated DNA exchange uses a lipid membrane-based nanoscale tube to bidirectionally share materials (e.g., DNA, proteins, and other biomolecules) between host and recipient as the connection links the cytoplasm of the adjoining cell^104^ (Figure 1E). Furthermore, unlike conjugation which relies on specialized conjugative plasmids and/or ICE containing DNA sequences, the genetic materials is not so specialized^107^ (Table 5). Furthermore, biological macromolecules other than DNA can be shared including RNA, proteins, and metabolites.^107^ In the Gram-positive bacterium like *B. subtilis*, special hydrolases (LytC-amidase and its enhancer (LytB) are employed in nanotube-mediated DNA exchange to penetrate the recipients cell wall barrier in an interspecies compatibility-dependent manner.^107^ Although this mechanism is regarded as a route of genetic exchange among neighboring cells,^88,104^ it also serves as a hallmark of the cell’s death phase due to biophysical stressors.^108^ Since the result of nanotube formation is the eventual death of the microbe, and subsequent release of the genetic material into the immediate environment, development of a strategy capable of mopping the cell debris including the DNA and proteins would be a cost-effective means of HGT control and reduced antibiotic dependence.

**Table 5:** Comparison of Conjugation and Nanotube-Mediated DNA Exchange

Feature	Conjugation	Nanotube exchange
structure used	sex pilus + type IV secretion system	membrane nanotubes
genetic requirement	conjugative plasmid or ICE	nonspecific
directionality	unidirectional (donor → recipient)	bidirectional
specificity	species-specific or plasmid-specific	broad, even cross-species
transfer type	primarily plasmids	plasmids, chromosomal DNA, proteins

#### 1.4.4 CTCNT-Mediated Plasmid Transfer (CTCNT-P)

Cell-to-cell natural transformation-mediated plasmid transfer (CTCNT-P) is a recently discovered mechanism involving the transfer of genetic information between strains of Bacillus bacteria.^109^ Although the mechanism has not been resolved, it is highly efficient compared to natural transformation and enhanced by cellular stress generated by antibiotic assault.^109^

## 2 HGT and the Global Spread of Resistance Evolution

Genes acquired via HGT enhance the fitness of the recipient organism and enable microbes to exploit novel ecological niches that would be inaccessible through standard mutational processes alone.^110,111^ Through the acquisition of a new genetic material, HGT generates new genomes from a common ancestor and results in distinct evolutionary trajectories.^112^ This evolution of resistance is sustained by continued selection through the exposure to antimicrobial drugs, usually at subthreshold or near subthreshold levels of effectiveness, and the emergence of new resistance strains of bacteria. Drug resistance is further perpetuated through the sharing of genomic regions that contain advantageous genetic traits with other organisms. There are several mechanisms of ARG that have evolved for the dissemination of drug resistance via HGT (Table 6). ARGs have long been established to be ferried by MGEs especially in the form of plasmids and transposons.

**Table 6:** ARG Mechanisms Spread by HGT

Mechanism	Description	Examples	Genetic elements driving global ARG spread via HGT
enzymatic inactivation	enzymes degrade or modify antibiotics.	TEM, SHV, mecA, and other β-lactamases, aminoglycoside-modifying enzymes	plasmids (Inc.F, Inc.I, and Inc.P), class 1 integrons, transposons (Tn3 and Tn4401)
gain of function mutation	spontaneous changes in chromosomal genes altering antibiotic targets or permeability.	rpoB (rifampin), gyrA (fluoroquinolones)	occasionally mobilized via transposons (e.g., Tn3)
efflux pumps	active expulsion of antibiotics from the cell, reducing intracellular concentration.	Tet(A), AcrAB-TolC	plasmid-borne efflux genes, integrons
target modification	alteration of antibiotic binding sites to reduce drug affinity.	erm genes (macrolides), mosaic PBPs	plasmid-mediated genes, transposons
target protection	proteins shield antibiotic targets without altering their function.	Tet(M), Tet(O), and Qnr proteins	plasmids, integrons, gene cassettes, and examples of innate antibiotic drug resistance

### 2.1 Enzymatic Inactivation

Genes encoding enzymes called the β-lactamases, which inactivate β-lactam antibiotics which include penicillin, cephalosporins, carbapenems, and monobactams, a process confirming resistance to beta-lactam antibiotics, have spread globally via HGT and thus drive many antibiotics into obsoleteness.^113,114^ Beta-lactamases fall into four main classes based on their catalytic mechanisms and structural features: Class A includes families like KPC, SHV, CTX-M, and TEM; Class B encompasses VIM and NDM; Class C includes ADC and CMY; and Class D comprises OXA-23 and OXA-48.^115,116^ All β-lactamases work through a well-characterized hydrolysis mechanism which is discussed at length in other reviews.^117–119^ For the sake of this review, we will focus on a few examples from the Class A beta-lactamase enzymes and their role in the emergence of extended-spectrum beta-lactamases (ESBLs). Beta-lactamases enzymes provide some level of innate immunity to their origin bacteria; TEM1 is from *E. coli* and the sulfhydryl variant (SHV) beta-lactamase was originally identified on the chromosome of *K. pneumoniae*.^120^

A great example of the impact at the global level for HGT to change and alter genomes can be observed with the bla_NDM_/β-lactam resistance gene. Initially, bla_NDM_, also known as the New Delhi metallo-β-lactamase/NDM gene, was characterized in *Klebsiella pneumonia* isolated from a Swede traveler returning from New Delhi, India, in 2008.^121^ However, prior to 2008, a bacterium *A. baumannii* carrying the bla_NDM_ gene was identified in an Indian Hospital in 2005.^121^ Subsequently in 2006, *Acinetobacter pitti* also tested positive for the bla_NDM_ gene.^122^ As mentioned earlier in this review, the bla_NDM_ gene is carried on a MRE which further demonstrates the effectiveness of HGT to enable the global trafficking of genetic information across different continents.^121,123–125^ The persistence of bla_NDM_ on a mobile antibiotic resistance gene has been disseminated globally throughout by both ICEs and transposons.^121^ The natural RP4 plasmid has been demonstrated to carry ARGs that provide tetracycline, ampicillin, streptomycin, and/or kanamycin resistance genes which are often transferred via conjugation.^126^ Despite the lack of publicly available complete genome sequences from the earliest observations, the initial bla_NDM_-positive isolates identified in 2005 were found to harbor the bla_NDM_ gene on multiple plasmids that were nonconjugative but possibly capable of transmobilization.^127^ Moreover, the initial isolates revealed the bla_NDM_ gene resides within a complete Tn125 transposon, accompanied by IS26 insertion sequences (ISs) and IS-containing common region 27 (ISCR27), indicating the potential for intricate mobility patterns since the gene’s initial integration event. Across various bacterial species, subsequent bla_NDM_-positive isolates consistently carry either a complete or fragmented version of the IS comprising Tn125 (ISAba125), located immediately upstream of bla_NDM_. The ubiquitous presence of ISAba125 in some form among all bla_NDM_-positive isolates identified to date, combined with early observations in *A. baumannii*, has led researchers to propose *Tn125* as the ancestral transposon responsible for the dissemination of bla_NDM_, with *A. baumannii* as the original host species harboring this genetic element.^128^

TEM1 is another beta-lactam that has spread globally (Figure 2); these enzymes are of concern because they enable the breakdown of cephalosporins like ceftazidime and cefotaxime. In the 80s and 90s, ceftazidime and cefotaxime were third-generation antibiotics which were effective against bacterial infections resistant to other beta-lactams.^130^ However, the emergence of specific mutations in TEM1, particularly G238S (Figure 2) and R164S, promoted the hydrolysis of third-generation cephalosporins like ceftazidime and cefotaxime. Over time, these mutations spread via HGT, leading to levels of clinically significant antibiotic resistance and are currently commonly found in extended-spectrum β-lactamases (ESBLs).^131–135^

Once bacteria have resistance to antibiotics through the expression of gain-of-function enzymes like the mutant G238S TEM1, these bacteria will accumulate additional changes—including as mutations in other chromosomal genes or altering the antibiotic’s target site (e.g., penicillin-binding proteins)—to strengthen resistance.^135^

Several variants of SHV and TEM have been transmitted by conjugative plasmids.^112^ Conjugative efficiency in the schematics of ARG dissemination is a function of the host rather than the genetic elements. The term generalist bacteria has been used to describe bacteria that live in multiple habitats.^136^ Plasmid-harboring bacteria within these taxa serve as reservoirs of multidrug resistance genes, which explains why clinically important antibiotic resistance genes such as mcr-1 and bla_KPC‑1_ are frequently detected on plasmids across human, animal, or environmental sources. Transposons and conjugative plasmids contribute majorly to the spread of mcr-1 and bla_KPC_ in multiple habitats. For instance, mcr-1 was initially mobilized on ISApl1 and subsequently stabilized on multiple distinct plasmids.^136^ These elements served as the fulcrum for both dissemination and persistence of these resistance genes. Insertion sequences (ISs) show a strong relationship with ARGs in the host genome, and this association appears to be shaped by antibiotic exposure levels. When antibiotics pressure is low, ISs tend to associate with non-ARGs, such as transposable elements, and virulence factors, a pattern reported in the genome of mycobacteria and Microcystis.^137^ In contrast, environments with high antibiotic exposure demonstrate tight linkage between ARGs and ISs. The genus *Klebsiella,* for instance, exemplifies this pattern where ISs shows a strong association with ARGs.^137^ These findings suggest that IS-mediated association of ARGs is a primary mechanism facilitating HGT, particularly in bacterial population experiencing intense antibiotic selection pressure. Equally important is the silent role integrons and transposons play in HGT. The architectural diversity of transposons particularly as it relates to its highly recombinogenic nature is a major factor enhancing persistence of AR genes in bacterial chromosomes. The enzyme that catalyzes the transposition of genes (which is inherently part of the genetic makeup of transposons—Transposases) modify bacterial genome structure and function by catalyzing diverse genetic rearrangement and forming transient cointegrate replicons that enhances genetic material exchange between different DNA molecules present in the same cell.^138^ By mobilizing chromosomal genes, transposable elements can incorporate them into newly generated transposable units residing on plasmids and or other mobile DNA, thereby promoting HGT between cells.^138^ Often, the cotranscription of transposases genes along with some downstream genes results in the activation of silent AR genes already present in the chromosome. For instance, the streptomycin resistance transposon Tn5393 can activate a downstream promoter-less tetracycline resistance gene, thereby abetting HGT among bacterial isolates.^138^ Antibiotic resistance genes on plasmids frequently rely on integrons promoters for expression,^139^ strengthening an interconnected regulatory cascade that enhances conjugative transfer and sustains HGT between bacterial populations. While plasmids and transposons have been established as conveyors of resistance cassette in bacterial niche, constitutively, integrons on the other hand have been the resistance police by monitoring and enforcing the phenotypic expression of resistance gene cassettes carried by plasmids and transposons.

### 2.2 Gain-of-Function Mutation

Gain-of-function mutation Shigellosis, a gastrointestinal disorder characterized by intense often bloody diarrhea, is caused by the bacteria of the genus *Shigella*.^140^ The four different species of *Shigella* bacteria—*Shigella sonnei*, *Shigella flexneri*, *S. boydii*, and *S. dysenteriae*—account for well over 200,000 deaths worldwide each year.^141^ The most notorious among the *Shigella* genus is *S. sonnei*, which has been divided into four genomic lineages and clades.^142^ Not long after the discovery of its link to numerous deaths, *S. sonnei* *quickly* developed multidrug resistance (MDR) to sulfonamides, ampicillin, streptomycin, and tetracycline.^143^ The MDR of *S. sonnei* was facilitated by various combinations of antimicrobial resistance (AMR) genes that were transferred horizontally. A recently characterized subset of the globally dominant genomic lineage 3 of *S. sonnei* has become resistant to ciprofloxacin. This resistance is facilitated by three mutations (gyrA-S83L, gyrA-D87G, and parC-S80I) present in the quinolone resistance determining region (QRDR) of the gyrA and parC genes. In addition, refs 144 and 145 reported that the second-line antibiotic azithromycin, third-generation cephalosporin, amino glycosides. and the extended spectrum beta lactam antibiotic Ceftriaxone have been added to its repertoire, making it to WHO’s extremely drug-resistant (XDR) cadre of priority organisms.

Colistin/polymyxin E is a critical last-resort antimicrobial agent employed for treating Gram-negative bacterial infections that are unresponsive to other available therapeutic options.^146^ Although colistin resistance was traditionally considered to be exclusively chromosomally encoded, the recent identification of mobile colistin resistance (mcr-1) genes in hospital and community settings worldwide has challenged this notion. The mcr-1 gene encodes a phosphoethanolamine transferase enzyme that catalyzes the addition of cationic phosphoethanolamine groups to lipid A, thereby altering the structure of the bacterial outer membrane.^147^ A pivotal moment in understanding the horizontal spread of colistin resistance occurred in 2016 when the mcr-1 gene was discovered on an Inc.I2 plasmid in an *E. coli* strain isolated from a pig in China,^148^ demonstrating the plasmid-mediated dissemination of this resistance trait. Notably, the mcr-1 gene has been found to co-occur on plasmids harboring other antimicrobial resistance genes, such as those encoding extended-spectrum β-lactamases^149,150^ and carbapenemases,^151,152^ representing a significant concern for the potential codissemination of multiple resistance determinants. The emergence of multiple mcr variants has been documented globally, spanning various bacterial species across all inhabited continents.^146^ These mcr genes, conferring colistin resistance, have been detected in a diverse range of microorganisms, including *K. pneumoniae*, *S. sonnei*, *Salmonella enterica*, and *E. coli*, and have been found to be harbored not only on plasmids but also integrated into bacterial chromosomes.^153^ In a recent study,^146^ the intracellular mobility of the mcr-1-containing transposon Tn7511 within the bacterial niche was unveiled. Their findings revealed the ISApl1-mediated transposition of the Tn7511 transposon, harboring the mcr-1 gene, into the chromosome of the *E. coli* DH5α strain. The swift dissemination of the mcr-1 gene exemplifies the intricate dynamics involved in the spread of antibiotic-resistance genes across multiple genetic levels, including plasmids, transposons, bacterial species, and bacterial lineages, highlighting the complexity of this phenomenon.

Many bacterial species have the innate ability to withstand the effects of a particular antibiotic, regardless of prior exposure, which is referred to as intrinsic resistance.^154,155^ In addition to intrinsic resistance, antibiotic resistance in bacteria can arise through multiple pathways, including alterations to the target molecule (via mutations or expression of alternative Penicillin Binding Protein-PBP), reduced permeability to drugs by downregulating the porins necessary for β-lactam entry into the cell (Figure 3), overproduction of efflux pumps that expel the antibiotics, and the synthesis of enzymes capable of modifying or breaking down the antibiotic compounds;^134,156^ this is refered to as acquired resistance.

The mobilized colistin resistance-1 (MCR-1) gene provides resistance against polymyxins, a class of polypeptide antibiotics that are regarded as the last line of defense in treating life-threatening infections caused by multidrug-resistant Gram-negative pathogens.^157^ This resistance gene was reported to coexist with ESBL on plasmids in Enterobacteriaceae.^149,151,158^ This intra/inter species dissemination via HGT has been linked to the spread of resistance evolution. In vitro studies have demonstrated that natural transformation of the penA gene can confer penicillin resistance to *Neisseria* species such as *Neisseria meningitidis*, *Neisseria cinerea*, and *Neisseria flavescens*.^159^ In a similar study, the genes parC and gyrA play a role in the transformation of fluoroquinolone resistance between *Streptococcus pneumoniae* and several viridans streptococcal species.^159,160^ *Salmonella concord* with lethal intercontinental reputation for bloody diarrhea have resistance for chloramphenicol, ceftriaxone, trimethoprim/sulfamethoxazole, azithromycin, and Meropenem. This resistance to these antibiotics has made this bacterium to develop pandrug resistance (PDR).^161^ The genotypic characterization of multidrug-resistant bacteria shows a dynamic modulation trait in response to treatment.^162^ For instance, multidrug-resistant *E. coli* isolates were responsible for the opportunistic infections in pediatric cancer patients in a children’s cancer hospital in Egypt.^162^ These isolates bearing multiple resistance genes employed a wide array of resistance mechanisms ranging from antibiotic target modification to antibiotic inactivation. This broad arsenal of defense mechanisms limits the choices of antibiotics that can be used in the treatment of bacterial infections. Resistance genes found in the *E. coli* isolates were horizontally acquired from other bacterial species such as *S. flexneri* and *S. enterica*.

### 2.3 Efflux Pumps

Efflux pumps are transport protein systems that facilitate the export of the material from the cell.^163–168^ The gain-of-function mutations have been demonstrated to be either overexpression mutations or the introduction of foreign gene into the microbe’s genome.^135^ Bacterial cells augment their multidrug resistance through a two-pronged strategy. First, they reduce the number of porins in their outer membrane (OM), thereby limiting the entry of drugs, and second, they deploy multidrug efflux pumps, especially the modular tripartite efflux systems^169,170^ (Figure 3). The role of such mutations, which impede the passage of substances through porins, has been extensively reviewed and highlighted in the literature. For instance, experiments involving lipid bilayers revealed that a ΔmspA *Mycobacterium smegmatis* mutant strain exhibited significantly lower channel activities compared to the wild-type *M. smegmatis*.^167^ Furthermore, target protection has evolved as one of two mechanisms by which bacterial pathogens resist the tetracycline class of antibiotics.

The development of target protection has emerged as a key mechanism utilized by bacterial pathogens to confer resistance against the tetracycline class of antimicrobial agents. This resistance strategy involves the acquisition and expression of genetic determinants that protect the antibiotic’s cellular target, typically the bacterial ribosome, from tetracycline binding and inhibition. Through the expression of specialized proteins that bind to or modify the tetracycline binding site on the ribosome, pathogens can maintain protein synthesis and cellular viability in the presence of these antimicrobial compounds. For instance, the phenotypic expression of disinfectant and antibiotic resistance genes in *Salmonella* spp was found to be conferred by class 1 integrons.^171^ This disinfectant and antibiotic cassettes qacEΔ1 and sul1, respectively, are located within the 3′-conserved sequences of this integron. The efflux pump encoding gene qacEΔ1 ^139^ and the cell division inhibitor encoding gene sul1 ^172^ are both located on the class 1 integron in *Salmonella,* suggesting the interconnectedness of the resistance mechanism. It is possible that multiple mechanisms of resistance may exist on a gene cassette carried and integrated by a single integron. These resistance mechanisms may have been acquired via exposure to multiple antibiotics and functions as alternative resistance routes.

### 2.4 Target Modification

Target modification in bacterial drug resistance occurs when the antibiotic’s binding site is structurally altered, reducing the drug’s ability to interact with its target. This change preserves the normal function of the target while preventing the antibiotic from exerting its effect. Common examples include methylation of 23S rRNA by erm genes, mutations in DNA gyrase (gyrA) for fluoroquinolone resistance, and altered penicillin-binding proteins in MRSA. These modifications can arise through chromosomal mutations or acquisition of modifying enzymes, making them a key mechanism of resistance. The epidemic of *Shigellosis* in hospital setting is perpetrated by the instrument of HGT.^142^ The evolution of this bacterial infection is accentuated by the conjugative transfer of azithromycin resistance genes mphA and ermB between sublineages of *S. flexneri* 2a and *S. sonnei*.^145^ The erm­(B) gene, which methylates 23S rRNA and confers macrolide resistance, has been widely disseminated via horizontal gene transfer on plasmids and transposons.^167,173,174^

#### 2.4.1 Target Protection

Target protection is a resistance strategy in which bacteria produce proteins that protect the antibiotic’s binding site, preventing drug interaction while allowing the target to function normally. Unlike target modification, this mechanism does not change the target but uses protective proteins to block antibiotic access. Common examples include Tet­(M) and Tet­(O), which protect ribosomes from tetracycline, and Qnr proteins that shield DNA gyrase from fluoroquinolones. These genes often originate in environmental bacteria and have spread widely via horizontal gene transfer. For instance, the tet­(M) gene, carried on conjugative transposons such as Tn916, has disseminated globally among Gram-positive and Gram-negative bacteria.^175^

## 3 Targeting HGT as a Strategy to Control the Spread of Drug Resistance

The rise of antibiotic resistance in bacterial populations has led to a worldwide crisis. The response has been manifold involving strategies that include increased surveillance and of ARG’s in bacterial populations in health care settings, development of advanced diagnostic tools to identify new outbreaks and rapid point of care monitoring patient infections, and the creation of antibiotic stewardship programs that mandate for appropriate use of antibiotics in various conditions in healthcare and agriculture.^176–178^ The rise of antibiotic resistance has also led to many widely used antibiotics becoming obsolete. In the wake of this loss, there has been a surge of research to identify new classes of antibiotics, new inhibitors of enzymes like beta-lactamases, bacteriophage therapies, antimicrobial peptides, nanostructured surfaces and other nanoscale antimicrobial materials, AI-driven drug design, and CrispR-based antimicrobial strategies.^179–189^ However, time is running out, current estimates for the lifespan of a new antibiotic drug is limited, and with the emergence of new pathogens and new forms of resistance, the timeline may be shorter. Another strategy of slowing the spread of antibiotic resistance is blocking the processes that spread genetic information among bacterial populations. Blocking or interfering with the mechanism of HGT will slow the spread of ARGs and may help in the treatment of aggressive pathogens that acquire resistance rapidly. Recently, inroads to this relatively new means of controlling AMR include blocking transformation using nanomaterials, identifying components of quorum sensing that limit HGT mechanisms, and interfering with conjugation using specialized bacteriophage.

### 3.1 HGT in a Biofilm Environment

Microbes like bacteria, while single-cell organisms, are most often found in the context of complex multicellular communities called biofilms enabling community members to survive harsh conditions, exploit resources, and cooperatively metabolically survive harsh environmental conditions. These structured multicellular colonies such as flocs, sludges, slimes, pellicles, marine snow, and microbial mats aid in gut homeostasis and environmental bioremediation.^190,191^ Bacterial cells are embedded at a high density within a cluster known as extracellular polymeric substances (EPS). Biofilm architecture plays a crucial role in dissemination of resistance genes especially when the emergent community architecture hinders donor cells from entering the regions of high cell density.^192^ The community biofilms provided make it easier for ARGs to spread compared to the planktonic state. The spread of ARGs in the biofilm niche can be mitigated with a nanoparticle cocktail (Figure 5). Depending on the type and composition, the nanoparticle possesses intrinsic properties to dislodge the biofilm.^193^

**Figure 5:**
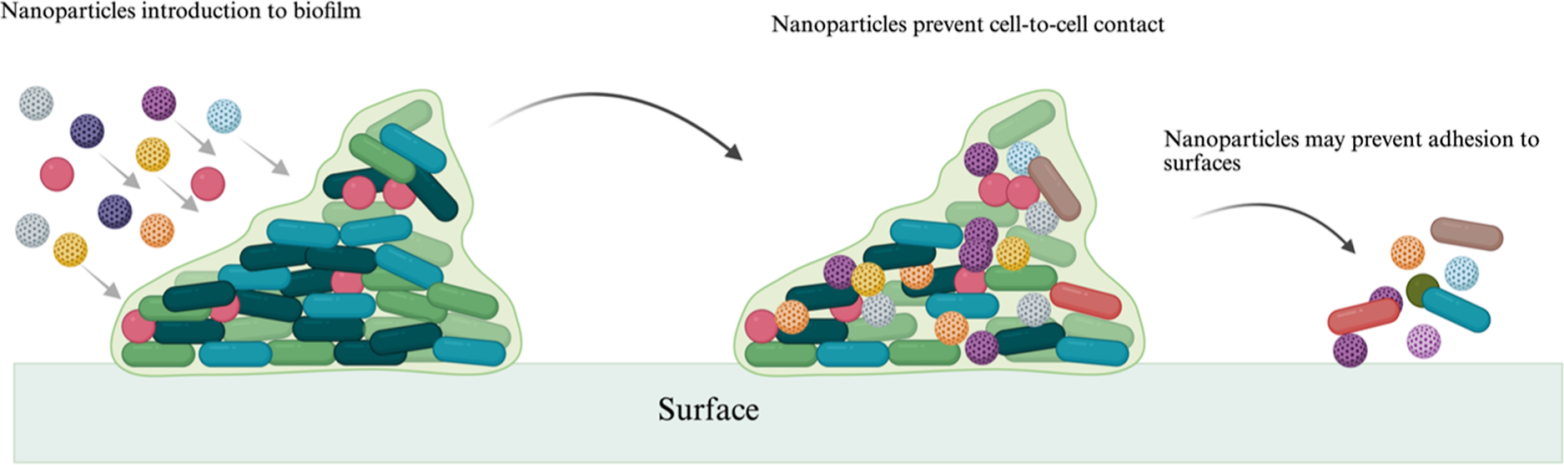
Proposed routes to control HGT. Nanoparticle disruption of the biofilm architecture, this essentially prevent cell proximity, thus preventing conjugation. In some cases, the nanoparticle may actively compete with the bacteria for the extracellular DNA. Sequestering the extracellular DNA is a route to limit resistance evolution.

Among the canonical routes in HGT, conjugative transfer is substantially enhanced within biofilm environments, enabling resistance genes to spread more rapidly and efficiently, indicating that biofilms not only enhance the spread of resistance genes but also increase the transfer rate by several thousands of magnitudes. The high cell density of cells within a biofilm may generate more friction resulting in dislodgement of bEVs, which constitute a huge reservoir of ARGs.^191^ In another instance, the conjugative plasmid—pGO1 conferring dual resistance to gentamycin and trimethoprim, shows an ∼16,000-fold increase in conjugative transfer efficiency in biofilm-dwelling compared to planktonic *S. aureus* presumable due to high cell density and altered transport within the biofilm matrix.^191^ *Campylobacter jejuni* F38011 showed a 17.5-fold increase in transformation frequencies of chromosomally encoded resistance genes in a biofilm compared to a planktonic cell.^194^ These two examples further underscore the critical role of HGT in biofilms, but it also reinforces the enhanced efficiency of genetic transfer in both HGT routes in the context of biofilms.

#### 3.1.1 Environmental Intervention

Detecting HGT in a natural environment remains a significant technical obstacle in microbiology. Recombination events arising from natural transformation and transduction are particularly challenging to identify because the acquired genetic elements integrate into conserved chromosomal loci. In a clinical environment, for instance, quantifying transformation and transduction frequencies is problematic since transferred DNA segments become embedded within genomic regions that are ubiquitous across all isolates of a given species.^195^ Environmental areas such as sewers provide opportunity for extensive contact between different environmental and human-associated bacteria creating a hotspot for HGT.^196^ Experimental approaches employing a fluorescently labeled genetic probe as bait DNA may facilitate the detection of transduction- and transformation-mediated DNA transfer events in clinical environments. Collectively, the pervasiveness of the HGT mechanism and the heterogeneity of the antimicrobial resistance determinant emphasizes the necessity for sustained, comprehensive investigations utilizing metagenomic methodologies.^197^

### 3.2 Targeting HGT as a Strategy to Control the Spread of Drug Resistance

The rise of antibiotic resistance in bacterial populations has led to a worldwide crisis. The response has been manifold involving strategies that include increased surveillance and of ARG’s in bacterial populations in health care settings, development of advanced diagnostic tools to identify new outbreak and rapid point of care monitoring patient infections, and the creation of antibiotic stewardship programs that mandate for appropriate use of antibiotics in various conditions in healthcare and agriculture.^183^ ^,^ ^203–205^ The rise of antibiotic resistance has also led to many widely used antibiotics becoming obsolete. In the wake of this loss, there has been a surge of research to identify new classes of antibiotics, new inhibitors of enzymes like beta-lactamases, bacteriophage therapies, antimicrobial peptides, nanostructured surfaces and other nanoscale antimicrobial materials, AI-driven drug design, and CrispR-based antimicrobial strategies.^184–194^ ^,^ ^206^ ^,^ ^207^ However, time is running out, and current estimates for the lifespan of a new antibiotic drug are limited, and with the emergence of new pathogens and new forms of resistance, the timeline may be shorter. Another strategy for slowing the spread of antibiotic resistance is blocking the processes that spread genetic information among bacterial populations. Blocking or interfering with the mechanism of HGT will slow the spread of ARGs and may help treat aggressive pathogens that acquire resistance rapidly. Recently, inroads to this relatively new means of controlling AMR include blocking transformation using nanomaterials, identifying components of quorum sensing that limit HGT mechanisms, and interfering with conjugation using specialized bacteriophage. Currently, several strategies are being developed: (1) eliminating ARGs in the environment, (2) blocking HGT by inhibiting critical cellular mechanisms, and (3) activating innate immunity in bacterial cells.

### 3.3 Eliminating ARGs in the Environment

Eliminating environmental DNA that contains ARGs involves both old and emerging technologies. Soil, water, and air are major sources of ARGs, and eliminating the availability of viable DNA reduces HGT, particularly in natural transformation; although damaging DNA will also impact other forms of HGT such as conjugation and transduction as well.^200,201^ The basic premise of these processes is to either destroy or damage DNA, thus preventing the transmission of viable information or sequester DNA and prevent DNA being available for HGT. These technologies include bulk approaches that to remove ARGs from the environment that do not involve a living component and cell/molecular based processes that target the mechanisms within the bacteria to prevent the spread of ARGs (Table 2).

#### 3.3.1 Bulk ARG Ablation Technologies

Abiotic technologies involve the application of DNA oxidizing or damaging agents including chemicals like chlorine, ultraviolet radiation, heat, and changes in pH.^202^ Many of these technologies are common practices and have been employed in a variety of settings including wastewater treatment and through composting of soil, mature, and wastewater slug.^211–213^ However, while these methods are widely applied, their effectiveness has been recently scrutinized and improved in the context of reducing the presence of ARGs in the environment. Recently, it has been demonstrated that composting an old and effective process of reducing bacterial pathogen also can unintentionally contribute to the spread of antimicrobial resistance.^204,205^ Researchers examined how antibiotic resistance genes (ARGs) and mobile genetic elements behave throughout the composting process and found that ARGs and ICE decreased during the high-temperature thermophilic stage but rebounded during the cooling phase due to chromosome-associated MREs that promoted horizontal gene transfer. Using mature compost, i.e., a fully decomposed, biologically stable organic material that has completed the thermophilic phase, improved control of antibiotic resistance by boosting thermophilic sterilization and reducing ARG hosts, ultimately preventing the rebound and increasing overall ARG removal by 8.3–14.9%.^206^ Furthermore, other recent studies have demonstrated that mature compost also reduces HGT in wastewater slug composting. The work demonstrates that even older technologies can be improved regarding the goal of reducing environmental ARGs.

Wastewater treatment is an important process for modern civilization and uses a variety of abiotic technologies to eliminate pathogenic bacteria from water.^207^ Wastewater treatment used several different processes including chemical and physical treatment of the influent, in addition to biotic treatments, which we will discuss later. Ultraviolet irradiation and chlorine have been demonstrated to be quite effective in reducing the concentration of environmental ARGs in wastewater treatment.^208,209^ Recent work has shown that a combination of UV and chlorine treatment resulted in the formation of chlorine oxide radicals which was responsible for the enhanced ARG degradation, while the other radicals (OH, Cl, and Cl_2_^‑^) played a minor role.^210^ This work demonstrated that improvement of traditional methods with a focus on targeting ARGS and HGT will slow the spread of drug resistance. Another promising method of ARGs reduction is a sulfate based on advanced oxidation processes. Recently, this method has been combined with other potent nonspecific oxidants such as hydrogen peroxide (H_2_O_2_) and ozone (O_3_) with a focus on wider efficiency with minimal detrimental environmental and economic impact.^211^ This method was effective in the degradation of resistant genes such as sul1, bla_TEM_, bla_OXA‑48_, bla_VIM_, bla_CTX‑M32_, intI1, and tetM in a wastewater environment.^211^ It is plausible that since the wastewater environment is one of the hotspots of resistance genes and bacteria, the application of AOP in biofilms in this area will undoubtedly remove resistance genes and bacteria.

#### 3.3.2 Membrane Filtration Technologies

Membrane filtration uses pressure-driven membranes to physically remove ARGs by size exclusion and electrostatic interactions, capturing both ARG-carrying bacteria and extracellular DNA.^212–214,217^ Microfiltration systems use several types of membranes, which use filters with larger pore sizes and is ineffective against free ARGs; however, ultrafiltration and nanofiltration or reverse osmosis are highly effective in removing ARGs.^223–226^ Membrane filtration limits ARG bioavailability—basically removing it from the environment—thereby reducing horizontal gene transfer. Advances in filtration systems include combining membrane filtration with photocatalysis. Recently, a filtrate with single cobalt atoms anchored on Ti3C2Tx provided dual reaction sites for efficient adsorption-degradation of antibiotic resistance genes.^216,218^ However, there may be issues with scale as increasing the volume through these systems often increases damage which reduces their efficiency to remove ARGs from water.^216^ Furthermore, while effective at removing organic compounds including antibiotics and clearing disease-resistant bacteria, larger systems including advanced peroxymonosulfate/solar processes which using a AOP process in conjunction filtration are not as effective at removing ARGs.^215^ These methods are more robust when combined with downstream or upstream treatments such as AOPs to address fouling and ensure genetic inactivation, and future developments may include embedding enzymes such as DNAase or other reactivity chemistries that degrade DNA on filtration systems to enhance removal of ARGs from water systems.

#### 3.3.3 Advanced Oxidation Processes

Advanced oxidation processes (AOPs) are treatment technologies that generate highly reactive chemical species, most notably hydroxyl radicals through the combination of UV irradiation with oxidants such as hydrogen peroxide, chlorine, or persulfate.^219–221^ The field emerged in the late 1970s–1980s as conventional treatment methods proved ineffective against trace, recalcitrant pollutants, with early development driven by drinking water and industrial wastewater applications. AOPs are now widely applied in advanced water reclamation, potable reuse, soil remediation from chemical contamination, and polishing steps for industrial effluents.^222,223^

AOP has not been used extensively in clinical settings due to some unique challenges including variable and complex wastewater composition, safety concerns surrounding the generation of toxic byproducts, and expense.^224^ Antimicrobial resistance (AMR) and virulence factors spread more quickly in clinical settings and the human microbiome HGT.^234^ ^,^ ^235^ ^,^ ^236^ ^,^ ^237^ HGT finds fertile ground in hospital environments, particularly through wastewater systems and sink drains that harbor antibiotic resistance genes and mobile genetic elements.^196,229^ Within these settings, biofilms serve as critical nexuses for gene exchange, with those colonizing medical equipment and surfaces proving especially conducive to rapid transmission.^190^ The gut microbiome presents another landscape where HGT thrives.^230,231^ The clinical consequences are profound: HGT-driven multidrug resistance escalates infection severity, mortality rates, and healthcare costs. *P. aeruginosa* exemplifies this threat, as its pathogenic capacity in lung infections hinges substantially on virulence and resistance genes acquired through horizontal transfer.^227^ ^,^ ^228^

AOPs are gaining traction in hospital wastewater treatment as an effective means to disrupt horizontal gene transfer of hotspots by targeting the genetic material itself.^232^ Recent innovations have expanded the toolkit considerably. UV-based AOPs represent a particularly promising advancement, with research showing that UV irradiation at 222 nm significantly outperforms conventional 254 nm wavelengths in inhibiting ARG transfer.^233^ This shorter wavelength damages both intracellular and extracellular DNA while reducing conjugation efficiency, making it well-suited for hospital water system disinfection. Building on this principle, UV/chlorine processes combine ultraviolet light with chlorine to generate reactive chlorine species that breach bacterial membranes and degrade resistance genes simultaneously. Comparative studies demonstrate that this hybrid approach achieves superior ARG removal relative to UV treatment alone, rendering it particularly effective for hospital effluents.^234^ Similarly, UV/hydrogen peroxide processes harness hydroxyl radical generation to oxidize DNA and compromise bacterial structural integrity, though their effectiveness is somewhat limited to extracellular resistance genes due to the radical scavenging capacity of intracellular components.^234^

Titanium dioxide-based photocatalysts and persulfate-driven systems demonstrate efficacy in degrading mobile genetic elements and reducing ARG reservoirs within wastewater treatment facilities.^235,236^ Another light-based AOP technique (photo-Fenton) was instrumental for the reduction in the spread of TetA and bla_TEM‑1_ genes in an *E. coli* model.^237^ Here, the authors used photo-Fenton process to generate reactive oxygen species that essentially degraded both intracellular and extracellular resistance genes. Even at a high dosage of 28 mg/L Fe^2+^ and 100 mM of H_2_O_2_, only a 0.5–0.8 log reduction in intracellular resistance genes was observed. This implies that the resistance gene may be latent even after the bacteria is wiped out. The effectiveness of the AOP process is not absolute, suggesting that transmission is still plausible. The report of Moreira et al.^238^ where qnrS, bla_TEM_, sul1, and intI1 showed an average log reduction of 1.0 after photocatalytic treatment confirmed the summation. The inability to completely degrade these intracellular resistance genes may be connected to protection offered by the cell wall of the host bacteria. In contrast, HGT was completely prevented in Bacillus with the treatment of Fe­(VI)/PMS in the presence of the reducing agent hydroxylamine.^239^ However, despite the 2-fold decrease in intracellular tetA, a small fraction of Bacillus cells (0.0566%) survived the treatment, suggesting that while as a short-term solution this method is effective, but in long term, it may result in the evolution of bacterial strains that are somehow resistant. Further improvements are necessary to enhance the effectiveness of intracellular gene degradation.

Ozone-based AOPs further expand the options available, as ozonation combined with UV or hydrogen peroxide enhances both DNA degradation and microbial inactivation, curtailing ARG persistence in wastewater.^240^ The cumulative benefit of these approaches lies in their dual action: AOPs not only inactivate bacteria but also degrade extracellular DNA, substantially reducing the transformation potential of the remaining genetic material. Collectively, these findings suggest that advanced oxidation processes can meaningfully reduce ARG persistence in wastewater and clinical effluents, effectively limiting the environmental reservoirs that perpetuate resistance. Beyond light-based systems, catalytic and photocatalytic approaches show considerable promise. AOP using sulfonated nanoscale zerovalent iron/peroxymonosulfate (S-nZVI/PMS) was used to halt conjugation and remove ARGs and antimicrobial resistance bacteria in a water environment.^126^ This AOP method generates sulfate radicals such as SO_4_^–^, with high redox potential, long half-life, and wide pH range. Conjugation was halted by the oxidant S-nZVI/PMS via the disruption of enzymatic metabolism (Figure 6A).

**Figure 6:**
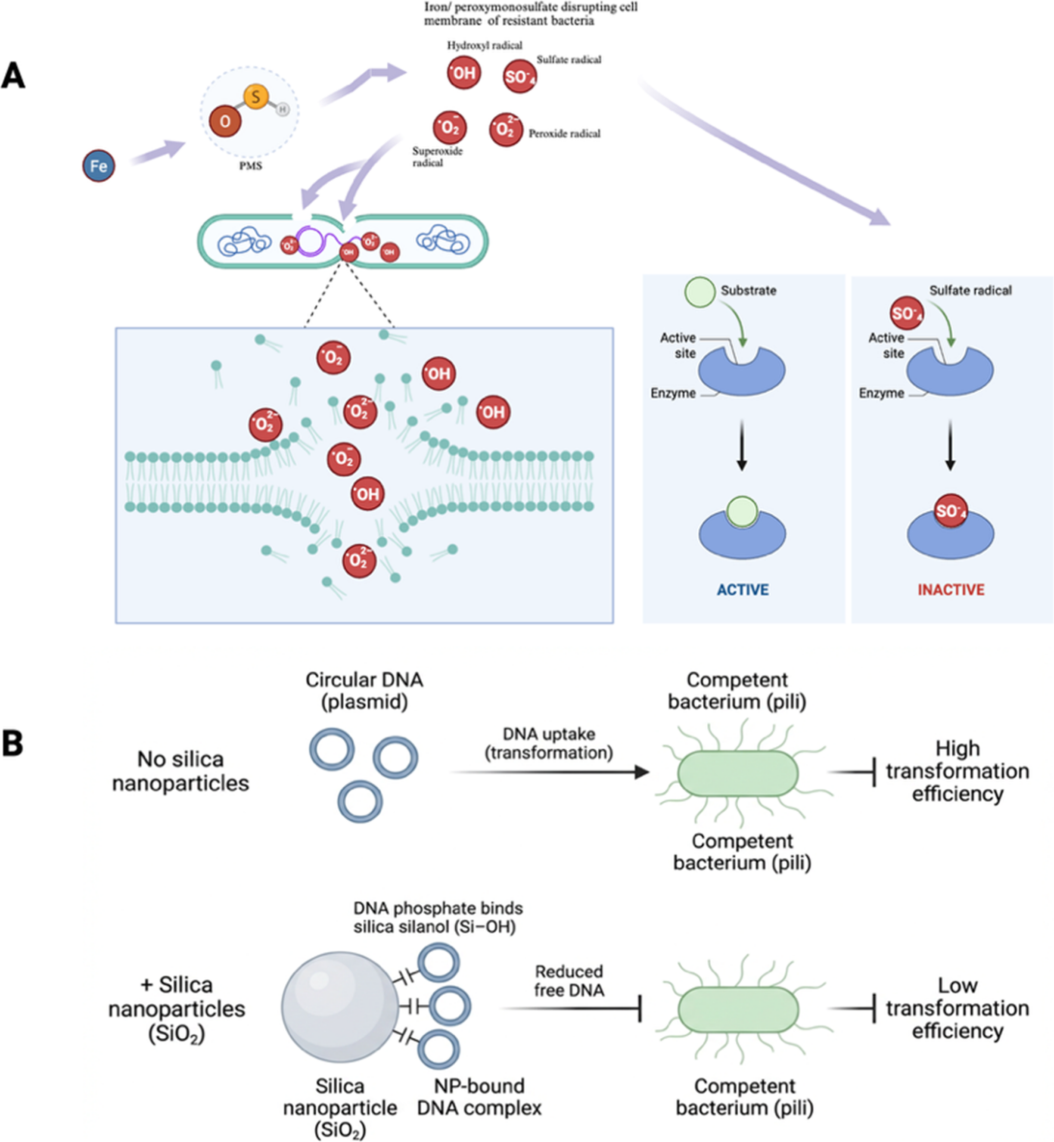
Proposed methods of controlling HGT in bacterial niche. (A) Iron activates the peroxymonosulfate (PMS) resulting to the production of radical species that degrade the cell membrane and disrupt enzyme metabolism, thus preventing conjugative transfer of resistance genes. (B) Silica nanoparticle binds to extracellular DNA by an attractive interaction between DNA phosphate groups and surface silanol groups on silica, thus sequestering the DNA and blocking transformation.

Another recent application for AOP technologies is the control of ARGs in soil environments. Soil environments often serve as reserviors for ARGs.^241–243^ Plasmid-mediated conjugative transfer of resistance genes has been reported between manure bacteria and soil bacteria.^244,245^ Soil-recovered transconjugants were found to possess amplicon sequence variants from the genera *Comamonas* and *Rahnella* which were plasmid transferred from organic manure, lending credence the effect of proximity in HGT events particularly between feces in manure and soil.^241^ Besides proximity, the mineral composition of the soil influences AR genes spread and subsequent evolution.^245^ Soil habiting bacterial genus such as *Listeria* harbors a huge repertoire of antibiotic resistance genes such as lin, norB, mprF, fosX, and sul which are mediated by transformation.^245^ The occurrence of transformation within the soil ecosystem provides an open-sourced opportunity to control the spread of resistance. Manures provide a unique environment for the propagation of HGT due to its nutrient dense nature, bacterial diversity, and a plethora of antibiotic resistance genes that may confer selective pressure on bacteria by triggering genetic mobilization.^244^

The application of AOPs to combat HGT in soil has promise. The AOP technologies using sulfonated nanoscale zerovalent iron combined with peroxymonosulfate (S-nZVI/PMS) could reasonably be adapted for soil environments. A straightforward approach would involve dispersing the treatment in water and applying it directly to soil surfaces. Encouragingly, studies have shown that this nanoscale iron compound significantly reduced intracellular resistance genes like intl1, tetA, and sul1.^219^ These methods might work equally well against extracellular resistance genes in soil ecosystems. A caveat with any AOP treatments is that they work by damaging bacterial cell walls, ultimately killing antibiotic-resistant bacteria through structural failure. This approach carries a significant hidden risk: when bacterial cells rupture, they release DNA fragments and vesicles that can fuel continued resistance evolution by creating “resistance gene hotspots” in the soil. Another drawback is chemical usage, which can present an adverse effect on plant metabolism such as toxicity. The use of a plant-derived catalyst might circumvent this challenge such as those derived from olive oil mills^246^ and food byproducts.^247^

#### 3.3.4 Absorption Technologies

Another strategy for the sequester of environmental DNA is to prevent its availability to transfer resistant traits to bacteria; these strategies are collectively known as DNA absorptive technologies. Absorption works with environmental DNA and therefore specifically interferes with natural transformation (Figure 6). There have been several different strategies employed including the use of absorbents, membrane filtration, and the application of cationic functionalized materials.^248–250^ Several different classes of materials have been used to bind and/or sequester environmental DNA. Adsorptive materials (e.g., biochar and certain metal oxides) strongly bind extracellular DNA, which reduces DNA mobility, immediate bioavailability, thereby suppressing natural transformation.

Biochar is a stable, carbon-rich form of charcoal that is generated through pyrolysis organic waste, such as wood waste, manure, or crop residues.^251^ The interaction of DNA with biochar is mediated through *p*–*p* stacking with contributions from the functional groups and doped material^252,253^ . These additional properties are in part due to the source of the organic material as well as the pyrolysis protocol. Recent work has demonstrated that biochar effectively removes environmental DNA and ARGS from both soil and water samples. Biochar has been used to reduce the concentrations of ARGs in the solid waste materials from antibiotic fermentation.^254^ Rice and corncob biochar were used to reduce ARGs from sludge vermicompost.^255^ Interestingly, biochar from different sources had different specificities on ARG with corncob biochar reducing the levels of the ermF and tetX genes and rice biochar reducing the levels of the sul1 and sul2 genes; the rationale for this is unclear, but possibly due to the presence of specific functional groups and levels of porosity or dopants. Cerium-modified biochar generated from activated sludge was used to reduce ampicillin resistance in solution.^250^ In this example, the cerium biochar eliminated the ARG not only through absorption but also through the generation of reactive oxygen species and persistent free radicles which in turn damaged the bound DNA. In a controlled study, biochar-activated peroxydisulfate reduced the titer of ARGs specially AbaF, tet by 0.87–1.07-fold.^256^ Porosity also is a critical structural feature of DNA absorbent biochar. Microporous biochar generated through the pyrolysis of cuttlefish bone exhibited an extremely high DNA binding compared to other adsorbents; this enhanced DNA binding which was more rapid was attributed to π–π bonding and large pore size.^257^ Biochar being a sustainable component of the circular economy being derived from waste material and diverse in composition and structure hold great promise as materials for removal of ARGs.^258^ Recently, nanobiochar was shown to repress ARG transduction in earthworm guts via a “phage shunting” mechanism through the conversion of lysogenic to lytic phages.^259^ More work is needed to determine what controls the properties of the biochar, especially biochar generated from different sources and how these properties such as surface functionalization and porosity can be modified to optimize the ARG removal process.

Silica (SiO_2_)-based materials have been used for decades to capture eDNA from water samples in laboratory and in real-world conditions.^260–263^ DNA adsorption to silica surfaces via electrostatic interactions is enhanced by the presence of divalent cations such as Mg^2+^ and Ca^2+^ through the formation of cationic bridges. Silica nanoparticles bind to both circular and single stranded DNA, respectively, via cooperative adsorption.^264^ However, the application of silica nanoparticles to remove environmental DNA and ARGs has only been done recently and under laboratory conditions. SiO_2_ nanoparticles reduced transformation efficiency by well over 90% in *Acinetobacter baylyi* ADP1 by sequestering a plasmid DNA^265^ (Figure 6B). The effect was dose dependent as increasing the ratio of nanoparticles to DNA enhanced the suppression of transformation. Moreover, changing the environment (i.e., the media) has had no impact on the transformation blocking effect. Interestingly, silica nanoparticles larger than 500 nm demonstrated a greater reduction of transformation, suggesting that surface area per particle is more important than the total available surface area. Many soils and clays are composed of similar metal oxides as these particles (e.g., SiO_2_ and alumina) and are also within this size range reported in this study. Although these experiments were performed in laboratory conditions, these results suggest that naturally occurring particles such as those found in sand and clays may be used to control the spread of ARGs in the environment; moreover, some soils have the capacity to bind DNA further strengthening this possibility.^266,267^

Absorption or sequestering of ARGs from the environment does not eliminate environmental ARGs; rather this genetic information is simply less available to the bacteria. A counter to this problem is to combine absorption with AOP. The combination of the two technologies reduces the potential weaknesses of either alone. An example of this hybrid technology is seen with the chitosan-carbon quantum dot/ZnFe_2_O_4_ nanocomposite system fabricated and tested for the removal of ARGDNA sequences (e.g., tetA, sul1, and bla_CTX_) in the simulated hospital wastewater flow system.^258^ The nanocomposite integrated multiple DNA binding mechanisms—within a single, recyclable material. The resulting material demonstrated a broad spectrum of targeting and destroying ARGs and the tetracycline drug with high capacity and efficiency. The platform was extremely robust and maintained a high level of performance even after 20 cycles of use. This example of combining absorption with AOP demonstrates the versality and of combining these methods and providing a sustainable solution for a growing problem.

#### 3.3.5 Cellular/Molecular Based Anti-HGT/ARGs Technologies

Absorptive and bulk technologies have been used extensively in a variety of contexts including wastewater treatment and soil remediation and are being implemented in settings such as hospitals; however, other environments preclude these applications. For instance, an ARG spreading within a bacterial population of an infected patient cannot be removed easily by AOP methods. However, classes of HGT control technologies that target the living systems directly are being developed and may be used in these circumstances. Furthermore, controlling at the cellular level may also find roles in larger scale bulk system as well. These new technologies involved controlling environmental ARGs and their spread by assaulting the mechanisms that cells use to engage in HGT. These technologies include using compound or materials like nanoparticles that activate endogenous cellular processes or eliminant certain MDR genes, employing competence blockers that inhibit HGT process, phage-based systems and genetic based technologies such as antiplasmid systems and gene drives. Some of these methods enable the inhibition of more than just natural transformation but also conjugation and transduction.

#### 3.3.6 Chemical/Material Interrogation of Cellular Processes Required for HGT

Controlling HGT using chemical compounds has been reviewed in Buckner et al., 2018.^268^ Attempts to “cure bacteria” by plasmids involve a variety of chemicals including detergents, biocides, DNA intercalating agents, antibiotics, psychotropic drugs, and plant-derived compounds (Figure 7B).^269^ The variability in action of some is a problem as the response to a curing compound can be variable. An example is the plant-derived compound plumbagin. In *E. coli*, plumbagin effectively eliminated a conjugative MDR plasmid and the RP4 plasmid from *E. coli* bacteria but only cured 14% of *E. coli* of a different plasmid pUK651, while effectively eliminating them from other pathogenic bacteria.^270,271^ These results suggest that the mechanisms that maintain plasmids in the cells differ enough for differences in the activity of these curing compounds. One of the challenges with the process of plasmid curing is that it is nonspecific and removes plasmids containing ARGs as well as plasmids carrying useful and advantages genetic traits. Moreover, there is no distinction between nonpathogenic bacteria and pathogenic strains. While the compounds have been demonstrated in the lab, large-scale testing remains to be validated. Another group of chemicals has been demonstrated to inhibit conjugation.^272,273^ Recent efforts have been made to identify conjugation inhibiting drugs that inactivate components of proteins involved in the plasmid transfer machinery (Figure 7A), specifically the Type IV Coupling Protein that links the relaxosome and the Type IV Secretion System (T4SS) using the computational approach.^272,274^ Metal and metal oxide nanoparticles have also been used as plasmid curing agents.^275–277^ Nanoparticles such as ZnO,^278^ TiO_2_, CuO, AIOOH,^279^ SiO_2_ and Au,^265^ and Ag^280^ have been applied to control the spread of antibiotic resistance.^5,237,280^ Sub-MIC platinum nanoparticles have been demonstrated to cure plasmids in *E. coli*.^277^ Under laboratory conditions, TiO_2_ was shown to reduce transformation efficiency by 31-fold.^279,281^ Exposure to nanoselenium at concentrations below 300 μg/kg, during composting, was demonstrated to reduce antibiotic resistance genes.^282^ Fe_3_O_4_@MoS_2_ nanoparticle complexes significantly reduced bacterial conjugation by disrupting of multiple genetic pathways.^275^ However, there are some caveats with these nanomaterial-based approaches as nanoparticles often induce environmental changes that enhance HGT.^276^ A problematic attendant effect is that some of these nanoparticles inadvertently continue the perpetration of resistance. HGT has been shown to be activated by ROS, sunlight, organic materials, and stressors, many of which are generated by nanoparticles. The literature often demonstrates these paradoxes: ZnO nanoparticles application to soil mesocosm showed remarkable HGT control by degrading resistance genes such as tetA1, tetB, sul1, sul2, aadA1, imp2, imp5, mefA, bla_CXT‑M_, and strB.^278^ In contrast, ZnO nanoparticles increase the expression of competence-related genes and enhanced transformation frequency by 1.8-fold.^5^ Regarding HGT, the effect nanoparticles make is a function of size, composition, the synthetic process, and the species of bacteria. Hence, some environmental conditions may elicit mitigation, while others may enhance HGT. Together, these interconnected inhibitory mechanisms created a comprehensive barrier to conjugative transfer, effectively suppressing the frequency of HGT between bacterial populations. Engineering of such nanoparticle complexes will ultimately accelerate the advancements in the development of mechanisms to mitigate HGT and by extension resistance evolution.

**Figure 7:**
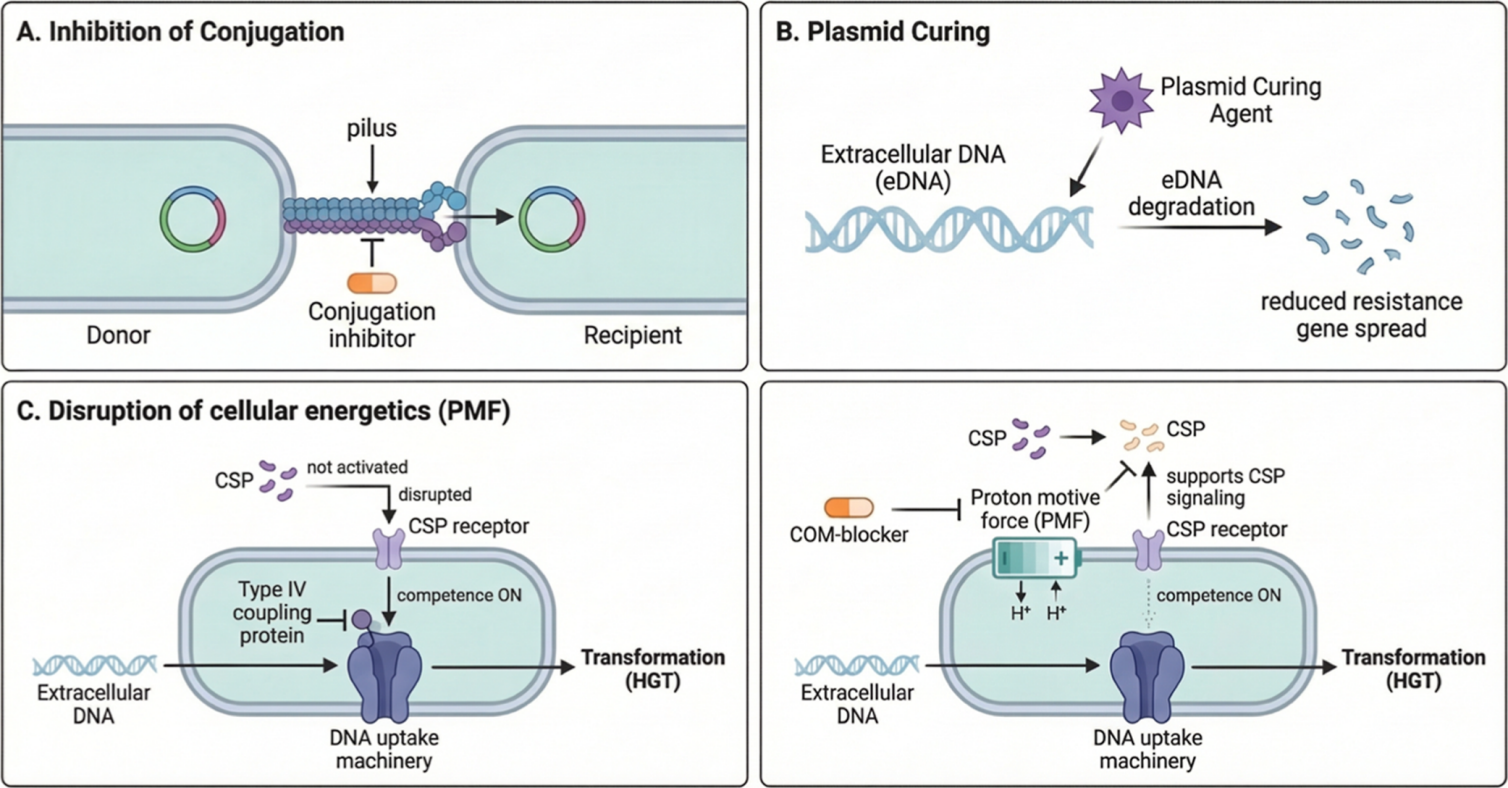
Strategies to control horizontal gene transfer. (A) Conjugation inhibiting drugs that inactivate components of proteins involved in the plasmid transfer machinery, specifically the Type IV coupling protein that links the relaxosome and the Type IV secretion system (T4SS). (B). Plasmid curing involves a variety of chemicals including detergents, biocides, DNA intercalating agents, antibiotics, psychotropic drugs, and plant-derived compounds that target eDNA for degradation. (C) Competence blockers (COM-blockers) disrupt the proton motive force, which inhibits quorum-sensing signaling via CSP, thereby preventing activation of the DNA uptake machinery.

#### 3.3.7 Competence Blockers: Antievolution Compounds

Quorum sensing is a microbial form of chemical cell-to-cell communication in bacteria that regulates gene expression based on population density.^85,283^ Through the release and detection of signaling molecules called autoinducers, bacteria monitor their local population size and synchronize group behaviors, such as biofilm formation, virulence, and bioluminescence, but only when a sufficient density, i.e., a “quorum”, is reached. The *Staphylococcus* spp. bacteria engage in a type of competence called QS-mediated competence which is controlled by an autoinducer known as the competence-stimulating peptide (CSP).^284^ Mutating the CSP peptide inhibits competence and synthetic competence peptides, stimulating protein1-E1A (CSP1-E1A), and competence stimulating protein 2-E1A (CSP2-E1A) competitively inhibit the ability of *S.* pneumonia to acquire the streptomycin resistance gene (rpsL) and the capsule gene cap3A during a mouse model of acute pneumonia and bacteremia.^284^ These works demonstrate that the CSP peptide serves as an excellent template to mutate and alter competence; CSP-based peptides are interesting targets that perhaps lend themselves to AI and machine learning tools to optimize for best competence blocking performance.^285^

In addition to attacking the CSP system directly, compounds called competence blockers (COM-blockers) have been identified that disrupt the proton motive force, which inhibits quorum-sensing signaling via CSP, thereby preventing activation of the DNA uptake machinery (Figure 7C).^286^ In a screen of over 1200 compounds, 46 were demonstrated to block transformation of *Staphylococcus pneumoniae*, a human pathogen that results in pneumonia and other respiratory infections. Several of the compounds were drugs used to treat other diseases identified including proguanil, an antimalarial drug; pimozide, an antipsychotic, and triclosan, a biocide; all were effective competence blockers at low concentrations (107). As “anti-evolution compounds, COM-blockers have great potential for controlling the spread of ARGs in a variety of contexts. By not being antibiotics, there is low selective pressure for developing against the blockers themselves. Although these need to be clinically evaluated; in the future, these agents may be used as adjuvants to current antibiotics or even treatment of waste water supplies, reducing the transfer of ARGs and reducing the emergence of multidrug-resistant strains in clinical settings. The ability to attenuate virulence and inhibit transformation in *S. pneumonia* by the addition of an exogenous compound demonstrates control over a process that would enable preventing the spread of resistance in many different contexts including infected patients. One limitation for COM-blockers is their specificity to the QS-competence of *Staphylococcus* spp bacteria and presumably other bacteria that use a proton motive to drive competence. However, other compounds have been demonstrated to control other forms of HGT. The anti-HIV drug AZT has been recently shown to inhibit HGT across a wide range of bacteria genera.^287^ AZT dissipated bacterial proton motive force, downregulated bacterial secretion systems, and inactivated thymidine kinase, which is associated with DNA synthesis, turned out to be the potential target of AZT.

#### 3.3.8 Activating Bacterial Innate Immunity to Combat HGT

The bacterial innate immune systems are composed of diverse, genetically encoded defense modules—often clustered in “defense islands”—including restriction-modification systems, Clustered Regularly Interspaced Short Palindromic Repeats-CRISPR-associated protein (CRISPR-Cas), Argonaut-based systems, cyclic-oligonucleotide signaling pathways (e.g., CBASS), and abortive-infection modules.^288–291^ These innate immunity systems detect nonself features of invading phages or plasmids, such as unmethylated DNA, foreign nucleic acids, or disrupted host mechanisms that are required for integration. Some mechanisms respond by cleaving invader genomes, blocking the replication of the foreign DNA, and/or triggering programmed growth arrest or cell death of the host cell (Figure 8A). Collectively, these mechanisms provide population-level protection by preventing the establishment and spread of mobile genetic elements.

**Figure 8:**
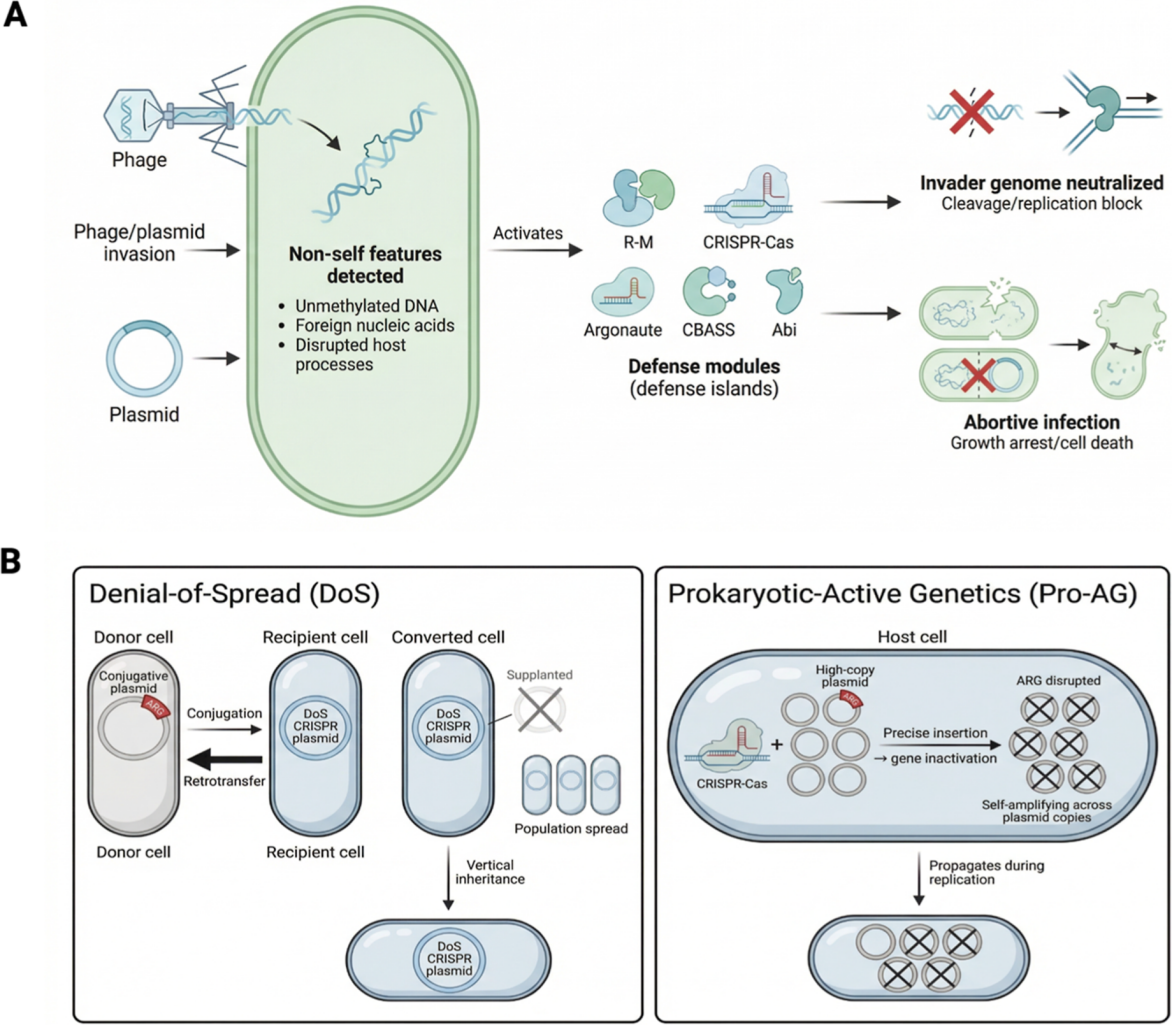
Bacterial innate immune defense against phages/plasmids. (A). Bacteria possess sophisticated innate immune mechanisms capable of mounting effective responses against invading phages and foreign genetic elements. These defenses comprise a diverse array of genetically encoded systems that are frequently colocalized within discrete genomic regions known as “defense islands”. The repertoire of characterized defense systems continues to expand and currently encompass restriction-modification systems, CRISPR-Cas machinery, Argonaute-based defense pathways, cyclic-oligonucleotide-based antiphage signaling systems (CBASS), and abortive-infection modules, each representing a functionally distinct strategy for detecting and neutralizing nonself-nucleic acids. (B). CRISPR-based prokaryotic gene drives targeting plasmid ARGs. Through this mechanism, the DoS plasmid is able to disseminate novel genetic information throughout a target bacterial population while simultaneously outcompeting the conjugative plasmid, displacing it not only as the primary vehicle of horizontal gene transfer but also as a heritable element passed vertically to daughter cells.

The CRISPR-Cas system is an adaptive immune mechanism prevalent in prokaryotes that prevents the acquisition of mobile genetic elements.^305–309^ ^,^ ^310^ Bacterial species with active CRISPR-CAS systems have been demonstrated to have fewer plasmids and prophage integration sites in their genome when compared to those that do not.^298^ Recently, activating the endogenous CRISPR-Cas system of bacteria has been explored to control HGT and the spread of ARGs. Bacterial defense systems exhibit synergistic antiphage activity.^299^ These mechanisms have evolved to counter bacteriophages and the introgression of foreign genetic into the host genome, so if these processes can be controlled to target ARGs, the HGT of drug resistance would be slowed or even stopped. In one study, the CRISPR system successfully inhibited the conserved conjugative transfer machinery used by plasmids and ICE.^298,300^ CRISPR-Cas systems counter HGT by providing sequence-specific immunity against foreign DNA, by targeting the foreign plasmids and integrative conjugative elements.^301^ The benefits of native CRISPR systems have already been demonstrated in clinical contexts. Active CRISPR-Cas mechanisms in *P. aeruginosa* diminished the acquisition of MREs, suggesting that these systems can effectively restrict HGT.^300^ In a recent study, Wheatley et al.^300^ reported the restriction of HGT by the CRISPR-Cas system in *P. aeruginosa*. While the primary target of CRISPR-Cas spacers is phages, a significant proportion, more than 80%, of *P. aeruginosa* isolates with an active CRISPR-Cas system contain spacers that specifically target integrative conjugative elements (ICEs) or the conjugative transfer machinery employed by plasmids and ICEs.^300^

#### 3.3.9 Engineered Crispr-Based Anti-HGT Technologies

While we have discussed CRISPR/CAS in the context of bacterial innate immunity, engineered forms of this system have transformed molecular biology, medicine, and biotechnology.^293,302,303^ At its core, Crispr/CAS systems are a diverse group of highly specific endonucleases that enable the targeting of any genetic element, DNA or RNA.^304,305^ Synthetic CRISPR constructs have been programmed to target conserved antibiotic resistance gene sequences, enabling plasmid curing and restoration of antimicrobial susceptibility in multidrug-resistant pathogens.^293^ In a computational model, CRISPR-Cas-encoding plasmids have been shown to restore antibiotic susceptibility in resistant bacterial populations by targeting plasmid-borne resistance genes despite genetic variation.^306^ In this model, the optimized DNA-cleaving/gene-silencing strategies for overcoming drug resistance depends on having a CRISPR bearing plasmid that is incompatible with the target plasmid and copy number. The model demonstrates that DNA-cleaving CRISPR-Cas systems are more effective than gene-silencing approaches when antibiotic resistance genes are chromosomally encoded because their removal ensures lineage extinction and a permanent loss of antidrug resistance genes (Figure 8A).

Recent innovations in the use of CRISPR-Cas9 systems to eliminate ARGs with a population involve nanoparticles or bacteriophage-based delivery vehicles, which selectively cleave resistance genes and plasmid backbones, thereby reducing conjugation capacity and compromising biofilm resilience.^307,308^ Bacteriophages vectors for CRISPR component enable precise delivery to target bacterial populations in both clinical infections and the gut microbiome.^309^ This bacteriophage-delivered approach facilitates a broader microbiome engineering strategy to disrupt biofilm architecture and restore antibiotic susceptibility across diverse bacterial communities.^310^ A conjugatively delivered, constitutively expressed CRISPR-Cas antimicrobial system has been demonstrated to selectively and efficiently eliminate antibiotic resistance genes from multidrug-resistant *E. faecalis* populations both *in vitro* and in the murine gut, offering a promising strategy for precision control of hospital-associated pathogens and engineered microbiomes.^311^ This study also extends to *in vivo* incidents where the CRISPR-Cas system was deployed in murine intestines to arrest the dissemination of the antibiotic-resistant *E. faecalis*.^292^ Hybrid systems that pair AOP with CRISPR-nanoparticle platforms demonstrate synergistic effects, with the chemical destruction of extracellular DNA occurring alongside the genetic targeting of intracellular resistance genes.^307^ This dual approach, chemical and genetic, offers a comprehensive strategy for dismantling resistance reservoirs and preventing their persistence. The recent discovery of pathogenic bacteria detection technique can be included as an adjuvant to the already discussed dual approach of AOP and CRISPR-CAS. Peng and colleague,^312^ using phage engineering, developed a reliable technique to detect pathogenic bacteria such as *V.cholera* and *P. aeruginosa*. This combinatorial method may be applied in hospitals and environmental settings in the following order:(1) detection, (2) AOP (to degrade extracellular resistance genes), and (3) Crispr-CAS (targeted degradation of intracellular resistance genes and resistant bacteria).

#### 3.3.10 Gene Drives

Gene drives are technologies that generate population-level genetic bias by spreading, enforcing, or eliminating specific genes; in the case of bacteria, this is through horizontal and intracellular mechanisms.^317^ ^,^ ^313–315^ These systems have been established in a variety of other structures including control of malaria mosquito populations, various eukaryotic genetic systems, and herpes virus.^316,317^ In microbes, gene drives are being adapted to block horizontal gene transfer by targeting and eliminating specific plasmids or ARGs (Figure 8B). Engineered CRISPR-Cas9 gene drive systems have been designed to block horizontal gene transfer function by targeting and destroying incoming antibiotic resistance genes or foreign plasmids inside bacteria, often reducing transfer efficiency by 2–3 orders of magnitude. These systems function as “predatory” gene drives that selectively eliminate harmful MREs while sparing beneficial microbes.^313^ By programming CRISPR-Cas9 to recognize specific resistance genes, gene drives reduce the risk that engineered or probiotic strains acquire unwanted traits through HGT.

One recently developed gene drive system uses an engineered, self-replicating plasmid, called the denial-of-spread (DoS) plasmid (Figure 8B).^315^ The DoS gene drive system exploits a weakness of the conjugation called retrotransfer in which genetic information travels in the reverse direction—from the recipient cell back to the original donor cell. This enables the DoS plasmid to corrupt a target population with this new genetic information and supplant the conjugative plasmid both in the horizontal transfer of genetic information and vertical inheritance. Once the conjugative plasmid is lost—this experiment by being outcompeted by DoS plasmids carrying Inc (iteron) sequences from the target conjugative plasmids—the DoS plasmid will be eliminated, leaving neither the target nor DoS remaining in the bacterial population. This enables the removal of target plasmid but maintain nontarget plasmids intact, thus reducing the collateral impact of eliminating all MREs from a bacterial population and the unforeseen consequences of such loss.

Another CRISPR-based gene drive strategy is known as Prokaryotic-Active Genetics (Pro-AG) which functions efficiently in a self-amplifying fashion similar to gene drive systems developed in diploid eukaryotes or in multicopy episomal herpesviruses.^314,318^ The Pro-AG systems disrupt ARGs carried on a high copy number plasmid by precise insertional target gene inactivation (Figure 8B). The Pro-AG gene drive system outperformed standard cut-and-destroy CRISPR^314,318^ anti-ARG gene drive by over 2 orders of magnitude. Unlike other anti-ARGs technologies of processes, gene drives are scalable and precise, eliminating only the target genes unlike alternative approaches that have been proposed that use chemical or nanomaterial targeting of MREs in general. Furthermore, the refinement of delivery systems of a gene drive system will enable applications in situations such as a patient where the delivery of a broader scale treatment with potential side effects may be less desirable.

##### 3.3.10.1 Antiplasmid Systems to Combat ARGs Spread

Antiplasmid technologies are intervention strategies that specifically remove, disable, or block plasmids, particularly those carrying antibiotic-resistance or virulence genes.^319^ antiplasmid systems often act locally within a cell or transiently within a population, but unlike other technologies, such as gene drives, they do not need to propagate through a population. Bacteria possess natural antiplasmid defense systems, including prokaryotic Argonautes (pAgos), DNA defense module (DdmDE) (Figure 8A), ApsAB, Wadjet, and Lamassu. ApsAB, pAgos, and Wadjet use nuclease and helicase or Argonaute-like proteins to detect and degrade resistance-carrying plasmids.^319,320^ The cellular processes degrade plasmids and bacteriophage DNA by blocking replication, direct degradation or removal of the foreign DNA from the cell. Recently, an antiplasmid system was demonstrated to be a promising therapeutic strategy to control antibiotic resistance in high-risk pathogens such as carbapenemase-producing *E. coli*.^321^ An antiplasmid-based system drives antibiotic resistance gene integration in carbapenemase-producing *E. coli* lineages. An advantage of antiplasmid strategies is their ability to restore the effectiveness of existing antibiotics. Because these approaches target the accessory genome rather than bacterial growth, they impose less selective pressure for resistance compared to traditional antibiotics.^322^

## 4 Conclusion

In this review, we cover the range of technologies that have been invented to slow the spread of HGT and ARGs from bulk methods like Ultrafiltration and AOPs that remove ARGs from soil and water to CRISPR-Cas and Gene Drive technology molecular techniques that change the ability of cells to access, assimilate, and express these traits. Much of how we frame these technologies is based on our understanding of HGT which until a decade ago focused solely on the canonical pathways of HGT—transformation, conjugation, and transformation. Anti-HGT technologies that clear ARGs from water or soil are designed to target ARGs that are carried by bacteriophage or in the form of environmental DNA. Many of the compound- or cellular-based approaches like COM-blockers or antiplasmid systems target mechanisms like conjugation. However, newer noncanonical modes of HGT, nanotube exchange, GTAs, or bEVs, may provide new challenges. Technologies like Gene Drives are versatile and assault the gene rather than the delivery system; AOP involves processes that eliminate all organic matter and probably eliminate ARGs contained in bEVs. Nanotechnologies provide new means of delivering and enhancing many of these technologies. We are at an impasse—on one side we have the rise of antibiotic resistance and on the other the challenge of finding new drugs to combat infection. Optimistically, we may be a stalemate; pessimistically, we have already lost. In either case, the edge has always been on the side of bacteria. They have been on this planet for a long time, and they know how to survive. Although we may be tittering on the brink, human ingenuity will always preserve, and the proof of this is in what we have accomplished so far.
